# Supplementary material for: Dynamic recurrence risk and adjuvant chemotherapy benefit prediction by ctDNA in resected NSCLC
Source: Nat Commun. 2021 Nov 19;12:6770. doi: 10.1038/s41467-021-27022-z (PMC8605017; doi:10.1038/s41467-021-27022-z)
Supplement: Supplementary file 1 — Supplementary Information [file 41467_2021_27022_MOESM1_ESM.pdf]

Supplementary Table 1. Recurrence-Free Survival Analysis by Clinicopathological Variables and Postsurgical ctDNA Status.

Character (*N*=85)	Univariate analysis[b]		Multivariate analysis[c]	
	HR (95% CI)	*P* value	HR (95% CI)	
**Gender**				
male vs female	1.4 (0.65-2.9)	0.42		
**Smoking**				
yes vs no	1.2 (0.57-2.4)	0.68		
**Age**				
<63 vs ≥63 yrs old	0.77 (0.39-1.5)	0.45		
**Histology**				
SqCC vs AD	0.78 (0.37-1.64)	0.52	0.44 (0.19-0.99)	0.047
other[a] vs AD	2.3 (0.52-10)	0.27	0.48 (0.08-2.9)	0.42
**pTMN stage**				
stage III/IV vs stage I/II	2 (0.96-4.2)	0.065		
**T staging**				
T4 vs T1-3	2.7 (1.1-6.5)	0.029	2.7 (0.95-8)	0.063
**N staging**				
N2 vs N0-1	1.6 (0.8-3.2)	0.18		
**TP53**				
mutation vs wild-type	3.3 (1.2-9.4)	0.026	4.2 (1.4-13)	0.01
**EGFR**				
mutation vs wild-type	0.98 (0.47-2.1)	0.96		
**Postsurgical ctDNA status**				
positive vs negative	4.0 (2.0-8.0)	3×10^{-5}	3.5 (1.7-7.4)	0.0007

AD: Adenocarcinoma
SqCC: Squamous cell carcinoma

[a] Large cell neuroendocrine carcinoma, adenosquamous carcinoma and atypical carcinoid
[b]Univariate Cox regression analysis
[c]Multivariate Cox regression analyses

p-Value was calculated by the log-rank test.

Supplementary Table 2. Recurrence-Free Survival Analysis by Clinicopathological Variables and Post-ACT ctDNA Status

Character (*N*=64)	Univariate analysis[b]		Multivariate analysis[c]	
	HR (95% CI)	*P* value	HR (95% CI)	*P* value
**Gender**				
male vs female	1.3 (0.51-3)	0.62		
**Smoking**				
yes vs no	0.83 (0.36-1.9)	0.66		
**Age**				
<63 vs ≥63 yrs old	1.3 (0.57-3.2)	0.51		
**Histology**				
SqCC vs AD	0.50 (0.19-1.3)	0.16	0.51 (0.19-1.4)	0.17
other[a] vs AD	2.37 (0.3-18.6)	0.41	3.8 (0.46-32)	0.22
**pTMN stage**				
stage III/IV vs stage I/II	2.2 (0.85-5.5)	0.11	1.6 (0.31-8.3)	0.57
**T staging**				
T4 vs T1-3	2.3 (0.79-6.9)	0.12	2.9 (0.59-14)	0.19
**N staging**				
N2 vs N0-1	1.9 (0.86-4.4)	0.11	1.9 (0.45-8.3)	0.38
**TP53**				
mutation vs wild-type	2 (0.59-6.7)	0.26		
**EGFR**				
mutation vs wild-type	1.1 (0.42-2.7)	0.9		
**Post-ACT ctDNA status**				
positive vs negative	3.2 (1.3-8.2)	0.009	4.4 (1.6-12)	0.004

AD: Adenocarcinoma
SqCC: Squamous cell carcinoma

[a] Large cell neuroendocrine carcinoma, adenosquamous carcinoma and atypical carcinoid
[b]Univariate Cox regression analysis
[c]Multivariate Cox regression analyses

p-Value was calculated by the log-rank test.

Supplementary Table 3. Recurrence-Free Survival Analysis by Clinicopathological Variables and Longitudinal ctDNA Status

**Character (*N*=89)**	**Univariate analysis[b]**		**Multivariate analysis[c]**	
	**HR (95% CI)**	***P* value**	**HR (95% CI)**	***P* value**
**Gender**				
male vs female	1.4 (0.67-2.8)	0.38		
**Smoking**				
yes vs no	1.2 (0.58-2.3)	0.66		
**Age**				
<63 vs ≥63 yrs old	0.83 (0.42-1.6)	0.6		
**Histology**				
SqCC vs AD	0.77 (0.37-1.62)	0.7	0.56 (0.26-1.2)	0.14
other[a] vs AD	2.42 (0.55-10.5)		0.87 (0.17-4.5)	0.87
**pTMN stage**				
stage III/IV vs stage I/II	2 (0.95-4.2)	0.068	1.6 (0.74-3.6)	0.22
**T staging**				
T4 vs T1-3	2.7 (1.1-6.5)	0.029	1.6 (0.57-4.7)	0.36
**N staging**				
N2 vs N0-1	1.5 (0.76-2.9)	0.24		
**TP53**				
mutation vs wild-type	3.2 (1.1-9.2)	0.027	2.2 (0.72-7)	0.16
**EGFR**				
mutation vs wild-type	0.9 (0.43-1.9)	0.79		
**Longitudinal ctDNA status**				
positive vs negative	8.5 (3.7-20)	2×10^{-9}	6.7 (2.8-16)	2×10^{-5}

AD: Adenocarcinoma
SqCC: Squamous cell carcinoma

[a] Large cell neuroendocrine carcinoma, adenosquamous carcinoma and atypical carcinoid
[b]Univariate Cox regression analysis
[c]Multivariate Cox regression analyses

p-Value was calculated by the log-rank test.

Supplementary Table 4. Performance of joint models and cox models

	Prediction time (months)	type	JM (value)	JM (value+slope)	JM (value+cumulative)*	Cox (Landmark)	Cox (Postsurgical)
AUC	12	training	0.89	0.86	0.90	0.73	0.75
		testing	0.84	0.83	0.89	0.70	0.71
	15	training	0.80	0.82	0.83	0.68	0.68
		testing	0.77	0.77	0.83	0.64	0.65
PE	12	training	0.13	0.12	0.11	0.11	0.11
		testing	0.14	0.14	0.12	0.14	0.15
	15	training	0.17	0.16	0.15	0.14	0.16
		testing	0.18	0.17	0.16	0.16	0.18

*JM (value+cumulative) was the final joint model used for comparison with Cox model

Supplementary Table 5. Targeted Panel Gene list

AKT1	AKT2	AKT3	ALK
APC	AR	ARAF	ARID1A
ARID2	ASXL1	ATM	ATR
ATRX	AXL	BIM	BRAF
BRCA1	BRIP1	BTK	CD274
CD74	CDA	CDH1	CDK4
CDK6	CDK8	CDKN1B	CDKN2A
CDKN2B	CHEK2	CREBBP	CTNNB1
CYLD	CYP2B6	CYP2C19	CYP2D6
CYP3A4	CYP3A5	DDR2	DHFR
DNMT3A	DPYD	EGFR	ERBB2
ERBB3	ERBB4	ERCC1	ERCC2
ERCC4	FAT1	FBXW7	FGFR1
FGFR3	FLT4	FRG1	GATA4
GNAS	GRIN2A	GSTM1	GSTP1
GSTT1	HDAC9	HGF	HRAS
IDH1	IDH2	JAK1	JAK2
KDR	KEAP1	KIT	KMT2A
KMT2C	KMT2D	KRAS	LRP1B
LZTR1	MAP2K1	MAP2K2	MED12
MET	MLH1	MTHFR	MTOR
MYC	NBN	NF1	NF2
NFE2L2	NOTCH1	NQO1	NRAS
NTRK1	NTRK3	PBRM1	PDCD1
PDCD1LG2	PDGFRA	PDGFRB	PIK3CA
PIK3CD	PIK3R1	PTEN	PTPN11
QKI	RAF1	RB1	RECQL4
RELN	RET	RHOA	RICTOR
ROS1	SBDS	SDC4	SETD2
SF3B1	SLC34A2	SMAD2	SMAD3
SMAD4	SMARCA4	SMARCB1	SOX2
STAG2	STAT3	STK11	TET2
TGFBR2	TP53	TPMT	TSC1
TSC2	TYMS	U2AF1	UGT1A1
VEGFA	WRN	XRCC1	

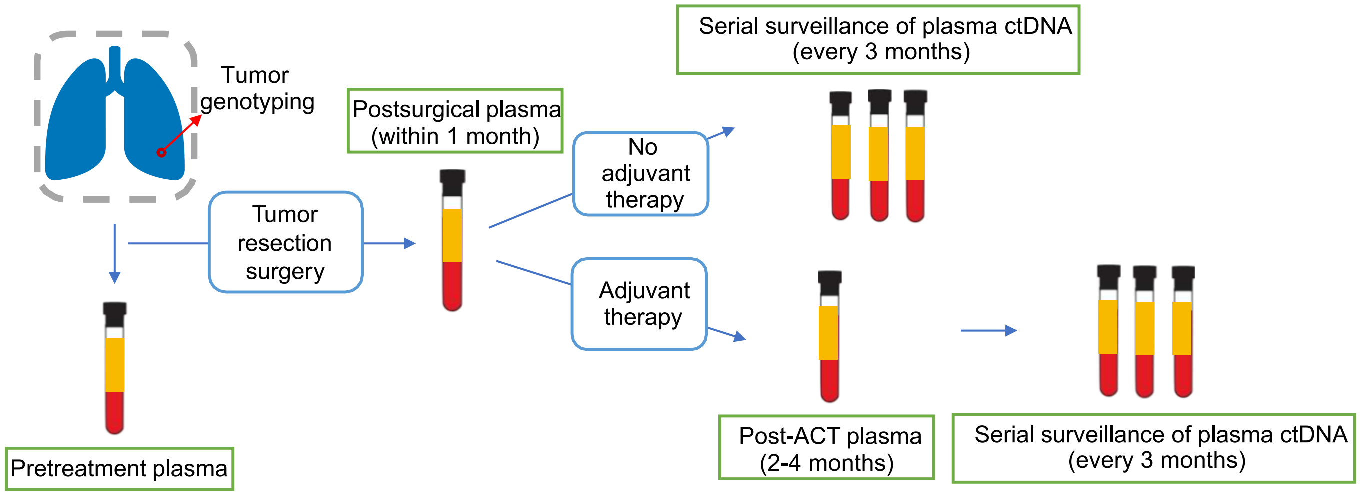

Supplementary Fig. 1 Tumor and plasma sample collection schedule.

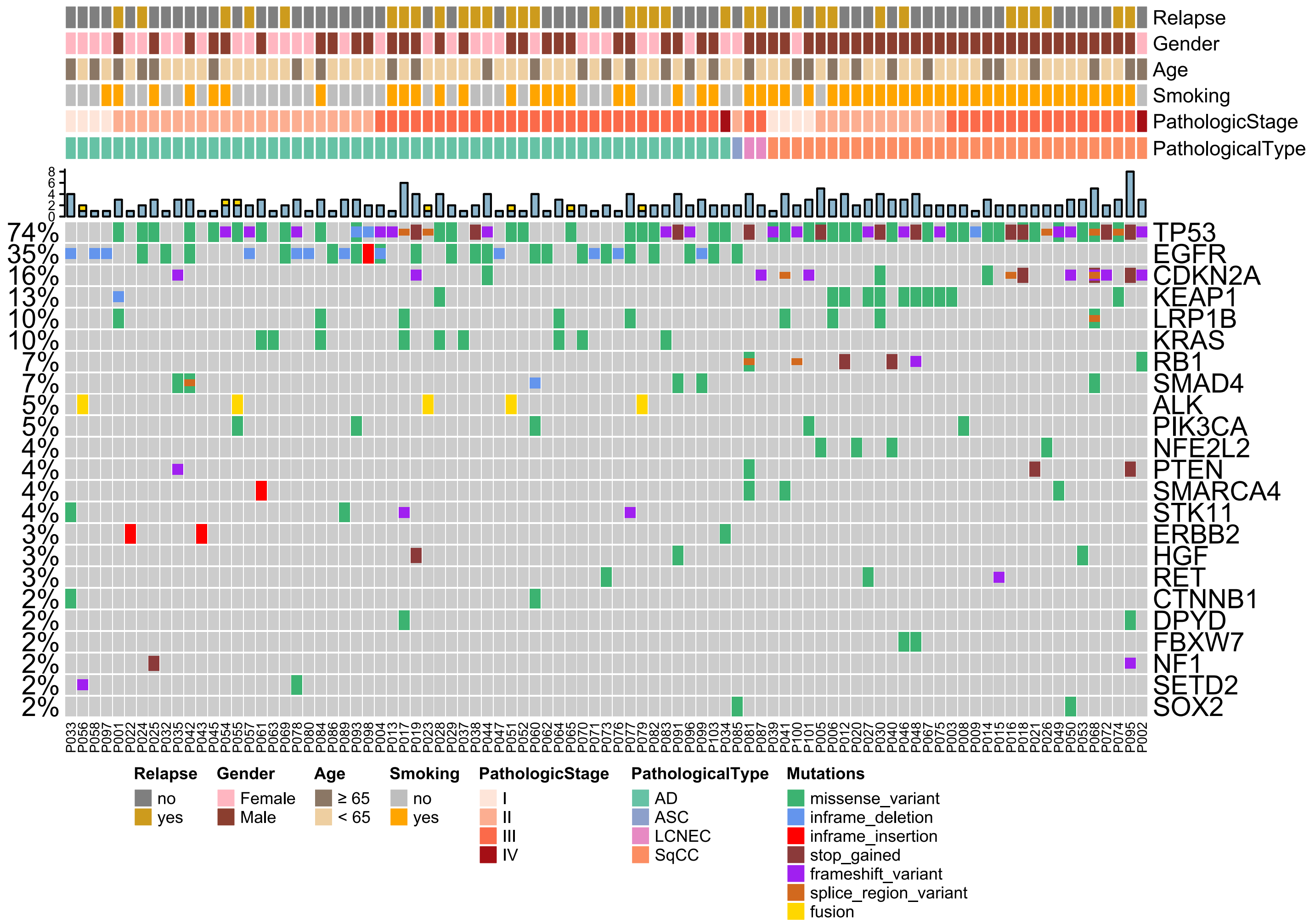

Supplementary Fig. 2 The mutational profile of tumor samples from 91 resectable Lung cancer patients. Each column represents one patient. The number of mutations in each patient was shown at the top, and the percentage of patients who had mutations in each gene was shown on the left. Only genes that were mutated in more than two patients were shown in the plot. AD, Adenocarcinoma; SqCC, Squamous cell carcinoma; LCNEC, Large cell neuroendocrine carcinoma; ASC, Adenosquamous carcinoma.

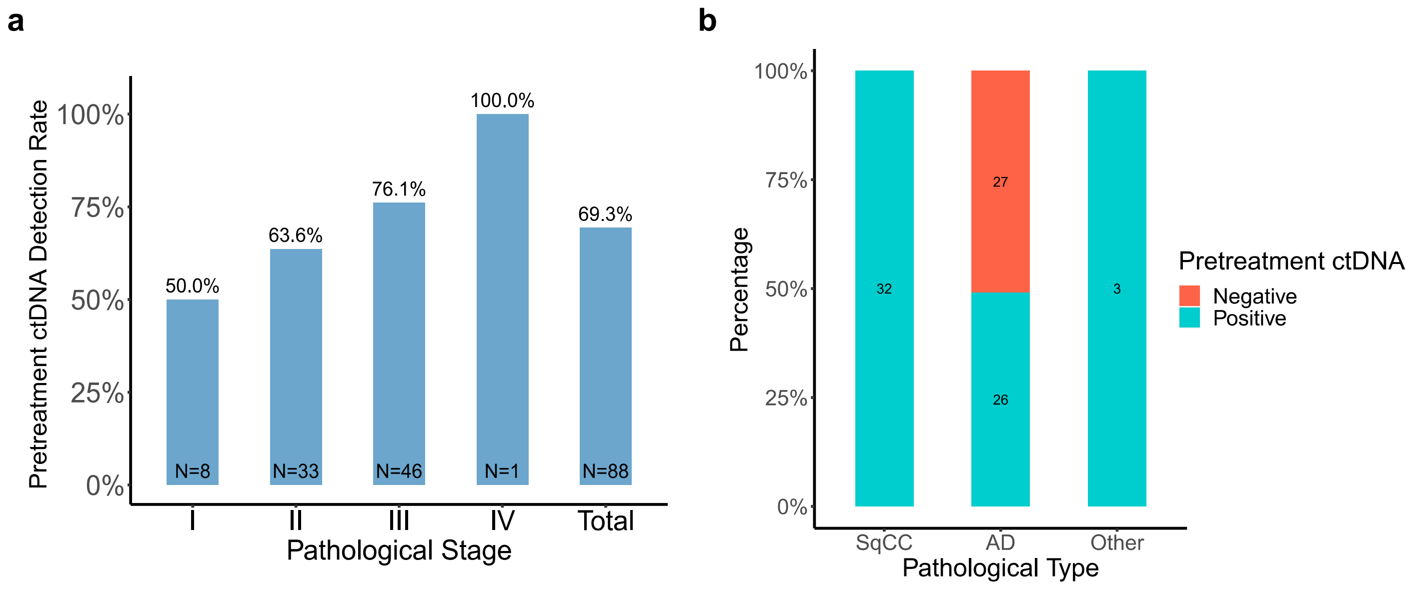

Supplementary Fig. 3 Pretreatment ctDNA shedding. **a** The proportion of pretreatment ctDNA-positive cases in different disease stages. **b** The percentage of different pretreatment ctDNA status in various histological subtypes. The specific number of patients in each subgroup was labelled within the corresponding column.

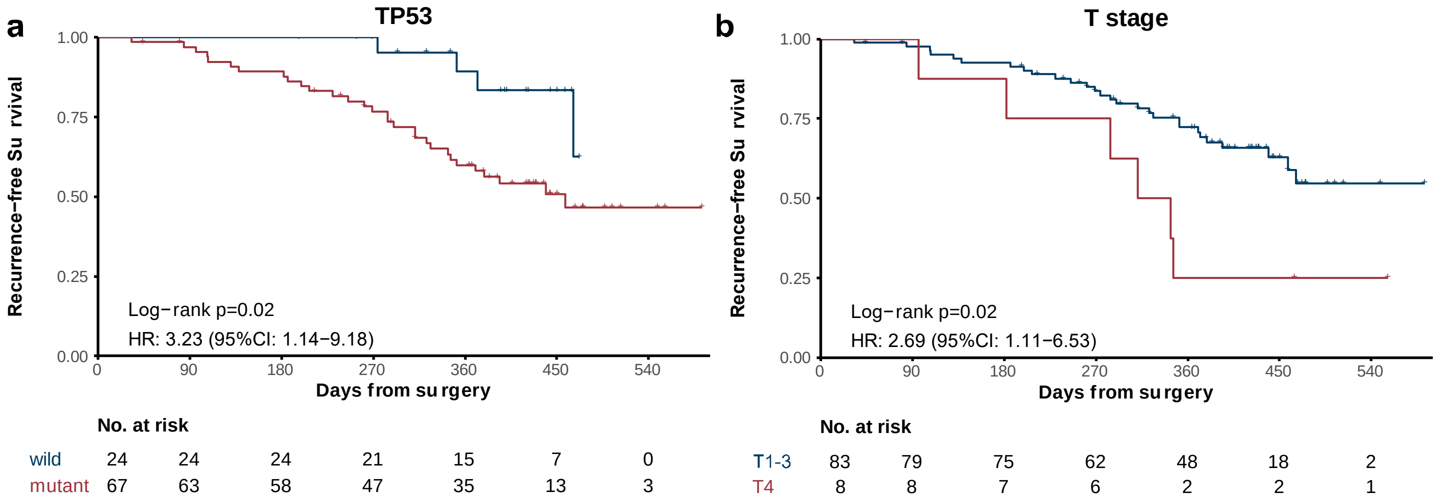

Supplementary Fig. 4 Kaplan-Meier estimates of recurrence-free survival (RFS) stratified by patient baseline characteristics. a Kaplan-Meier curve of *TP53* mutant vs. *TP53* wild type patients. **b** Kaplan-Meier curve of stage T1-T3 vs. stage T4 patients. Statistical significance was measured using Cox proportional hazards regression analysis. p-Value was calculated by the log-rank test.

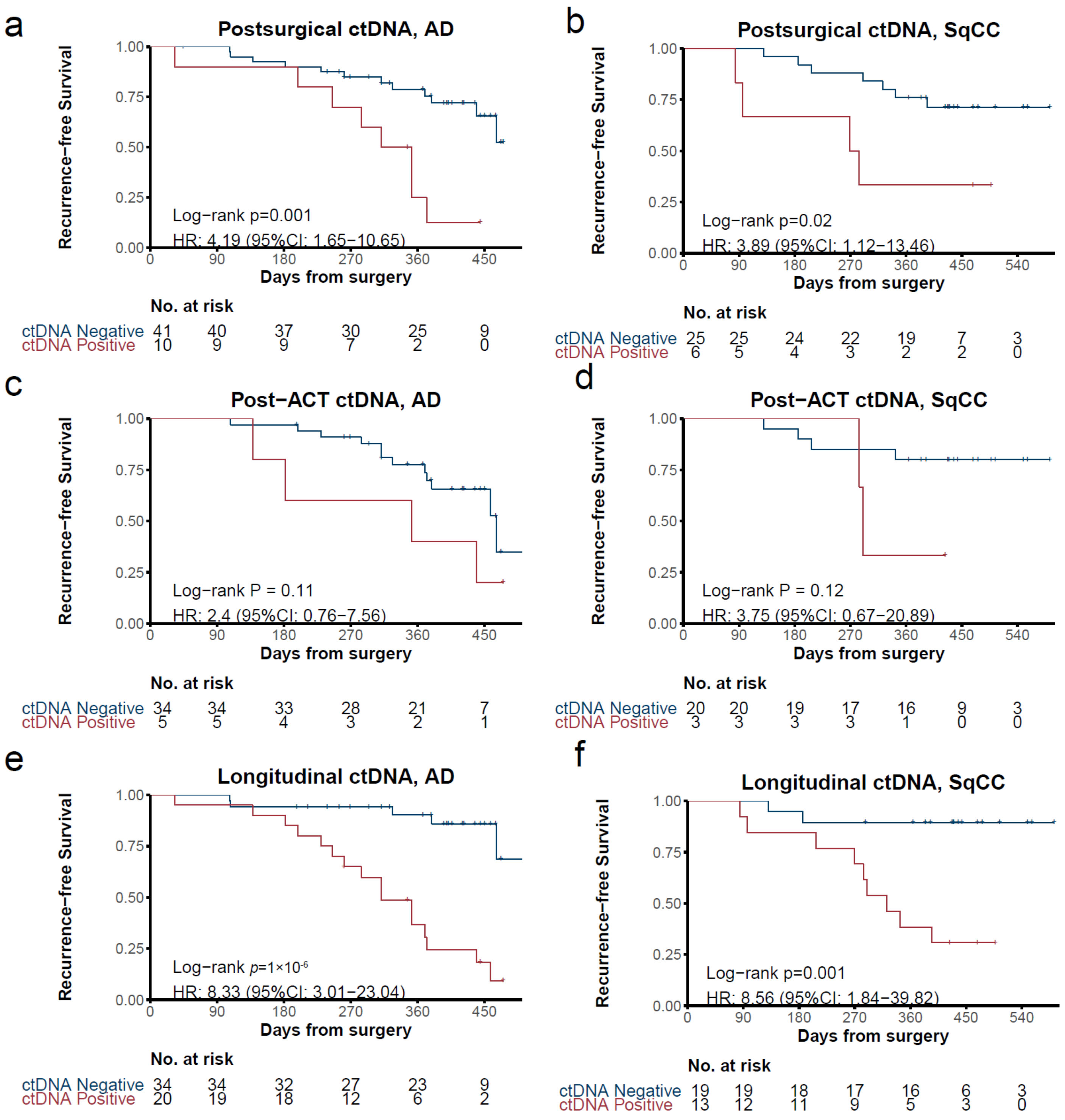

Supplementary Fig. 5 ctDNA positivity was related to patient prognosis in both adenocarcinoma (AD) and squamous cell carcinoma (SqCC) patients. a-b Kaplan-Meier curve of RFS stratified by postsurgical ctDNA status in AD patients (**a**) and SqCC patients (**b**). **c-d** Kaplan-Meier curve of RFS stratified by post-ACT ctDNA status in AD patients (**c**) and SqCC patients (**d**). **e-f** Kaplan-Meier curve of RFS stratified by longitudinal ctDNA status in AD patients (**e**) and SqCC patients (**f**). p-Value was calculated by the log-rank test.

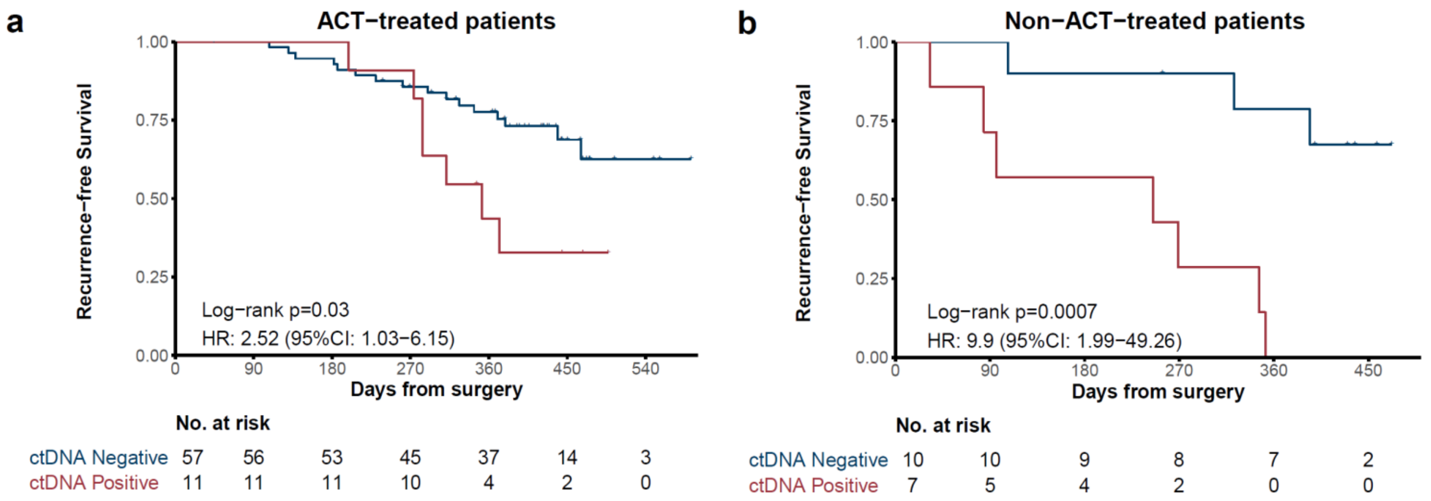

Supplementary Fig. 6 Positive postsurgical ctDNA was associated with worse RFS regardless of patients' ACT treatment status. Kaplan-Meier estimates of RFS stratified by postsurgical ctDNA status in patients with ACT (**a**) or without ACT treatment (**b**). p-Value was calculated by the log-rank test.

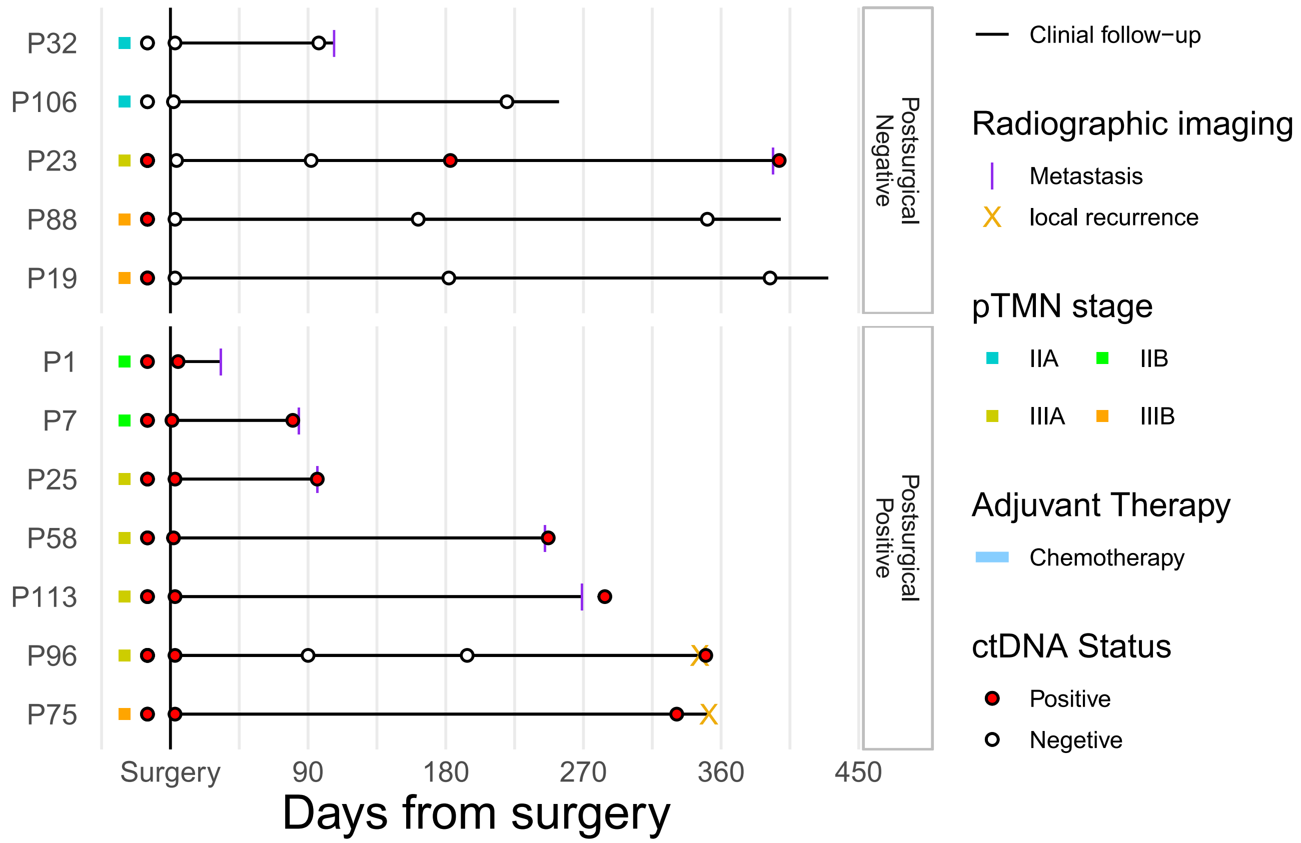

Supplementary Fig. 7 Recurrence status of postsurgical ctDNA-positive patients who did not receive ACT.

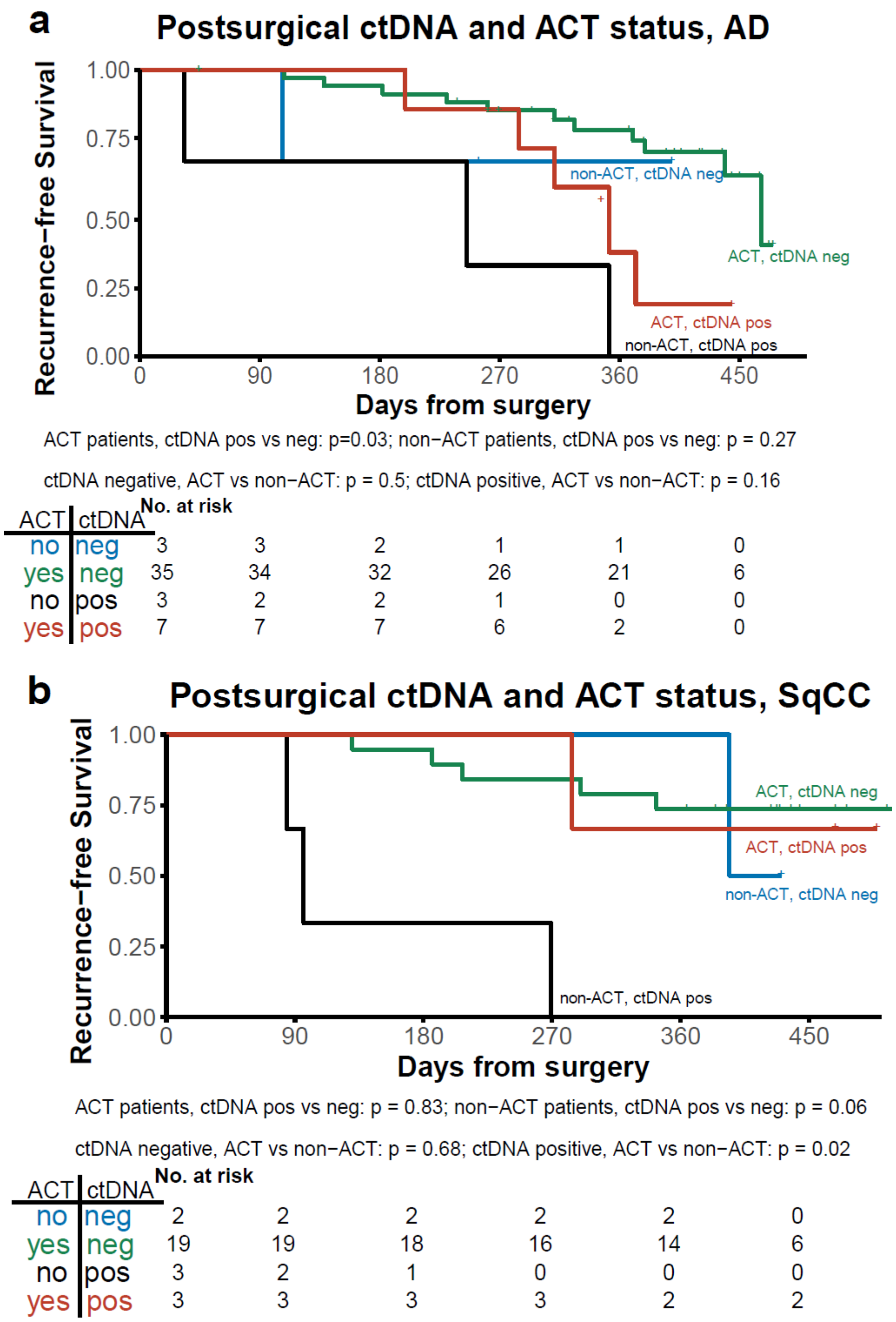

Supplementary Fig. 8 Kaplan-Meier curve of RFS in stage II-III patients stratified by both ACT treatment and postsurgical ctDNA status in AD (a) and SqCC (b) patients. Statistical significance was measured using Cox proportional hazards regression analysis. p-Value was calculated by the log-rank test for each comparison without adjustments.

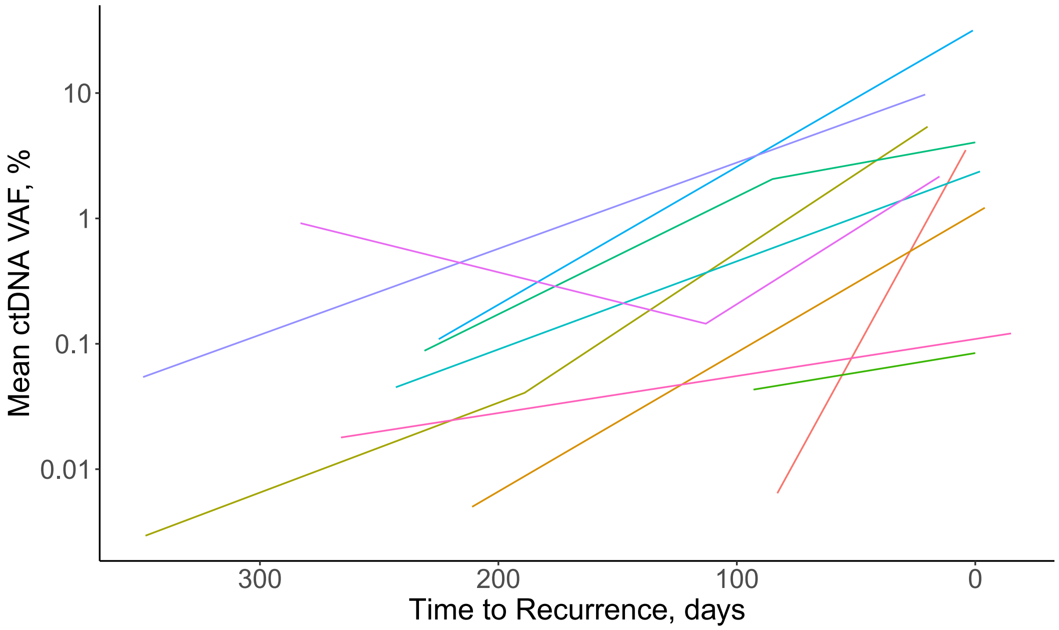

Supplementary Fig. 9 ctDNA variant allele frequency (VAF) changes from the first positive ctDNA detection to recurrence. Each colored curve represented data from a different patient.

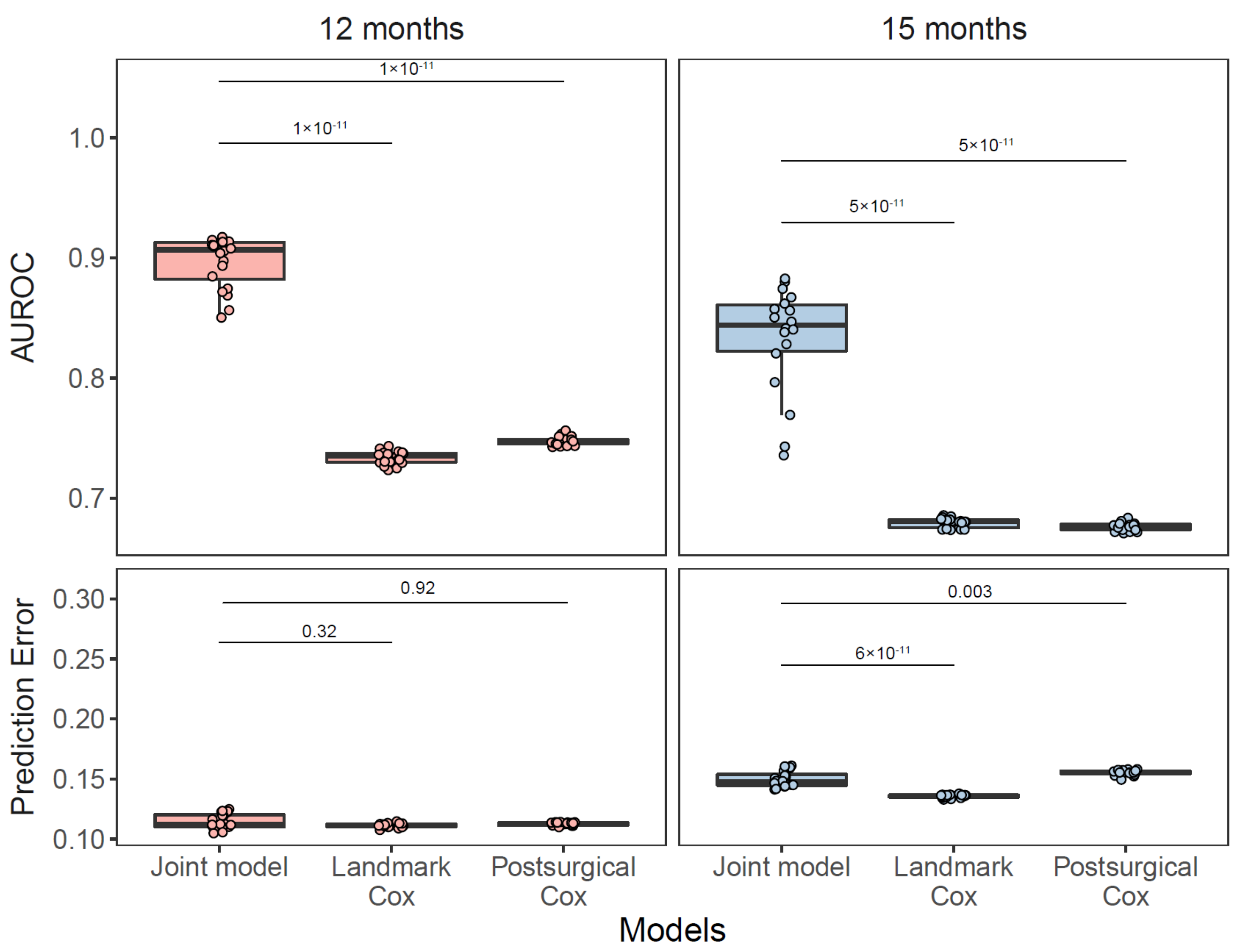

Supplementary Fig. 10 The comparison of model performance of training datasets between the joint model and cox models. Five-fold cross validation were repeated for 20 times. Time-dependent areas under the receiver-operating characteristics curves (AUROC) and prediction error (PE) represent discrimination power and calibration of the models. The p value is calculated using two-sided Wilcoxon signed-rank test. ns: not significant; *: p<0.05; **: p<0.01; ***: p<0.001. Center line, median; box limits, upper and lower quartiles; whiskers, 1.5x interquartile range.

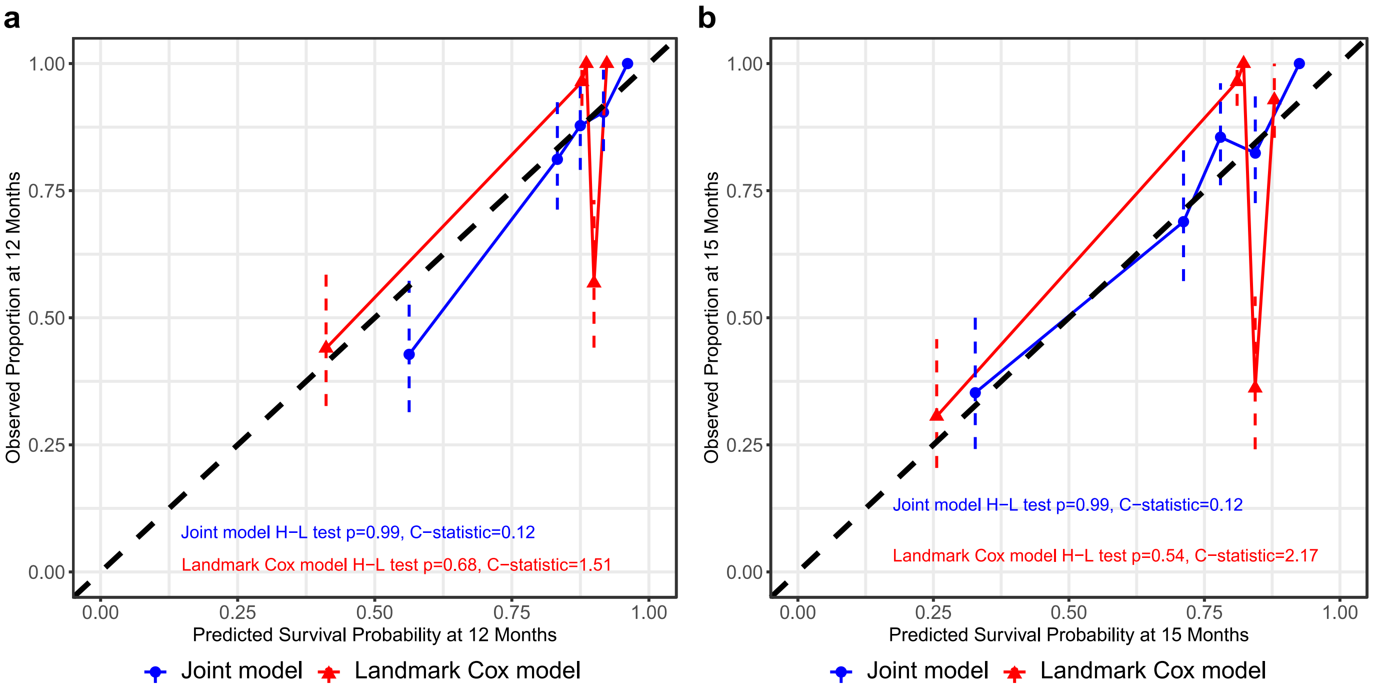

Supplementary Fig. 11 Reliability diagrams of the joint model and landmarking cox model.

Reliability diagrams of 12-month (**a**) and 15-month (**b**) estimates produced by joint model and landmarking cox model grouped for the Hosmer-Lemeshow (H-L) C-statistics. Data points of estimates produced by the models and their actual binary outcomes are plotted to show the distribution of the actual data. The number of patients within each bin is the same. Dashed vertical lines indicates 95% CI.

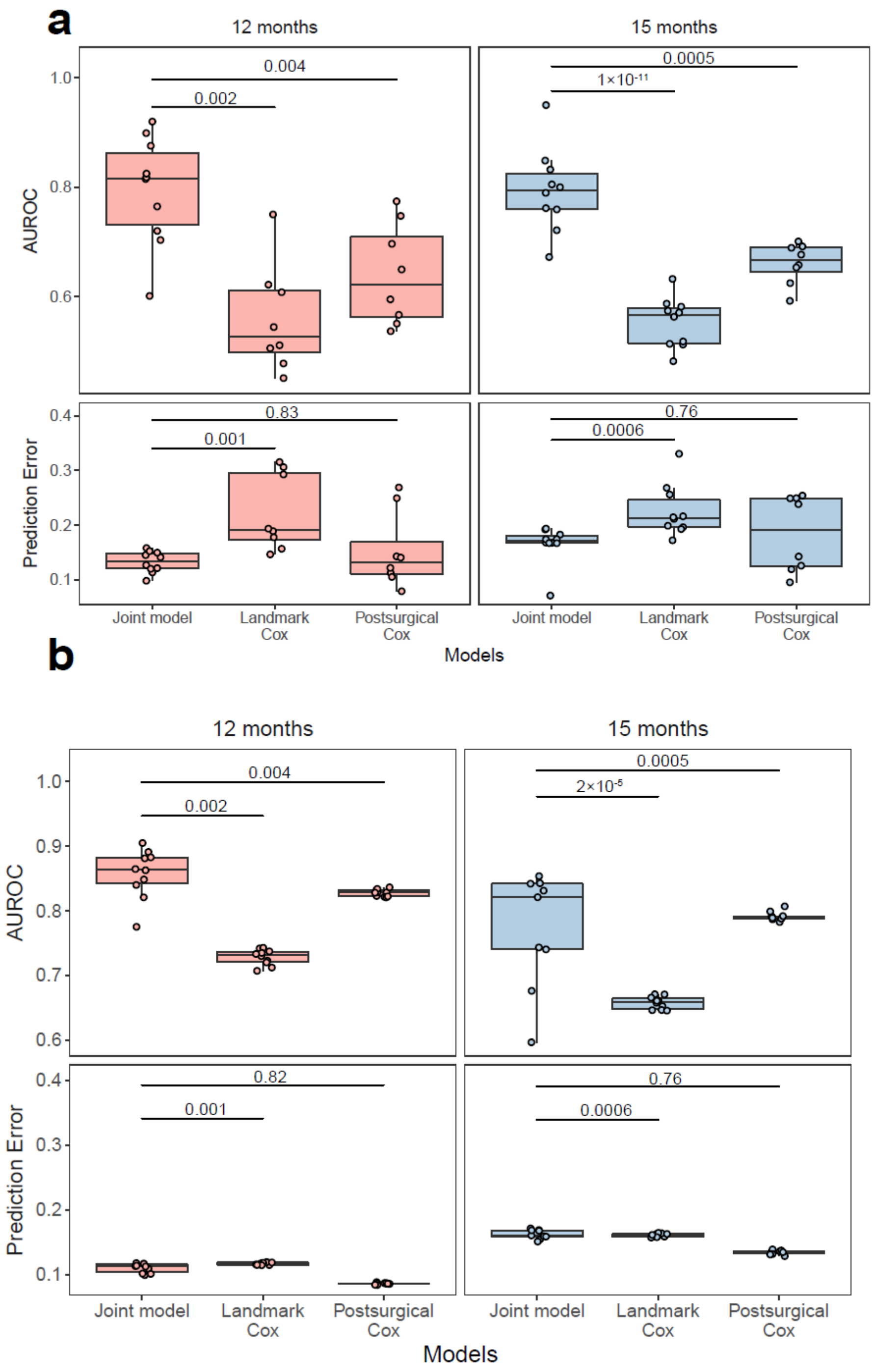

Supplementary Fig. 12 The comparison of model performance between the joint model and cox models using only AD patients. a Testing dataset. **b** Training dataset. Five-fold cross validation were repeated for 10 times. Time-dependent areas under the receiver-operating characteristics curves (AUROC) and prediction error (PE) represent discrimination power and calibration of the models. The p value is calculated using two-sided Wilcoxon signed-rank test. ns, not significant; *, p<0.05; **, p<0.01; ***, p<0.001. Center line, median; box limits, upper and lower quartiles; whiskers, 1.5x interquartile range.

P002, Relaspe-free

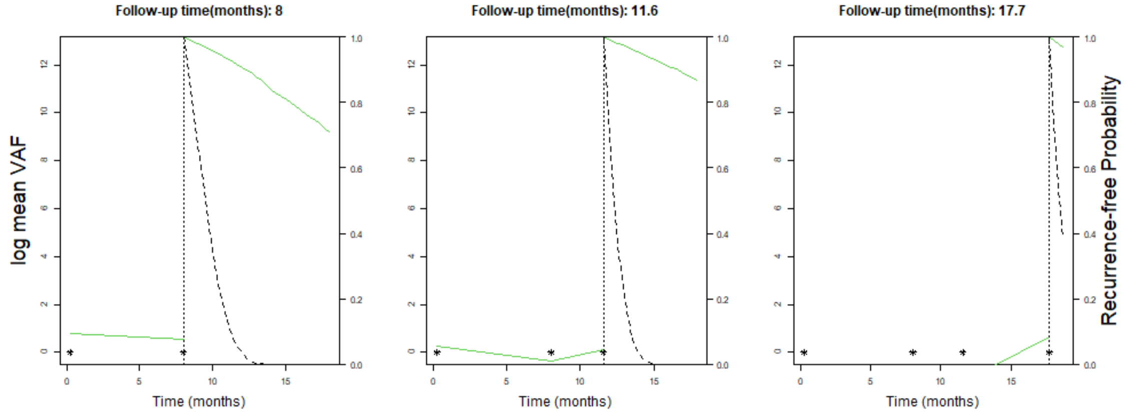

P003, Relaspe-free

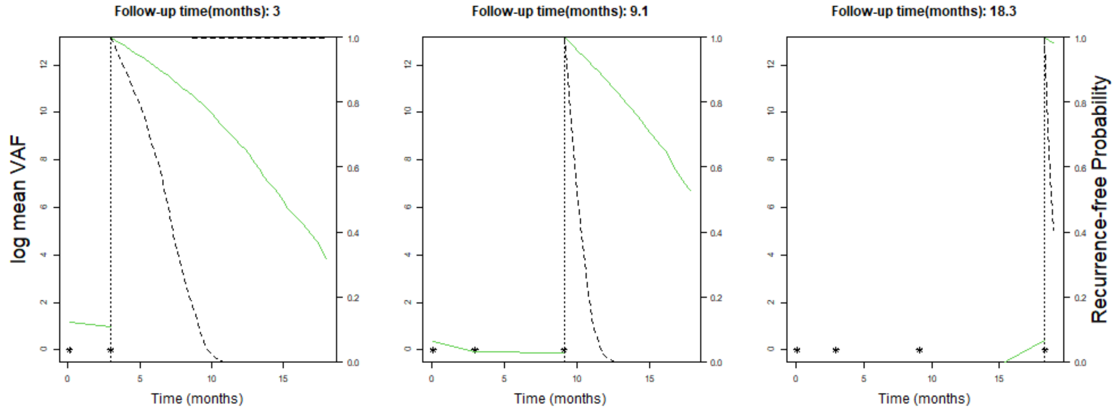

P004, Relaspe-free

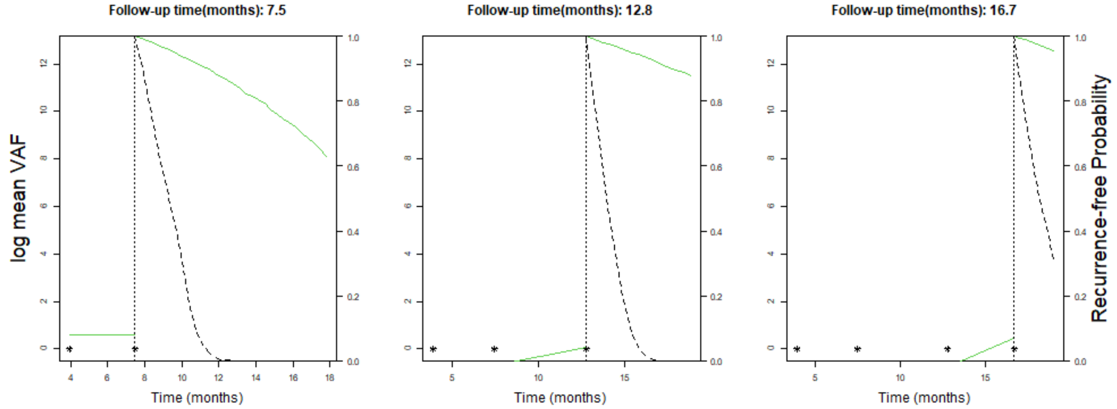

P005, Relapsed

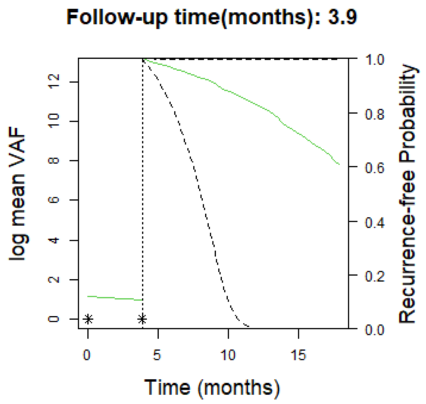

P006, Relapsed

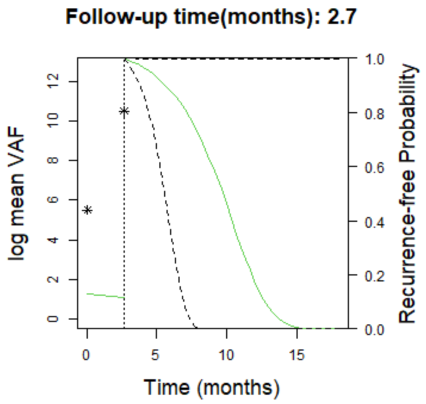

P008, Relaspe-free

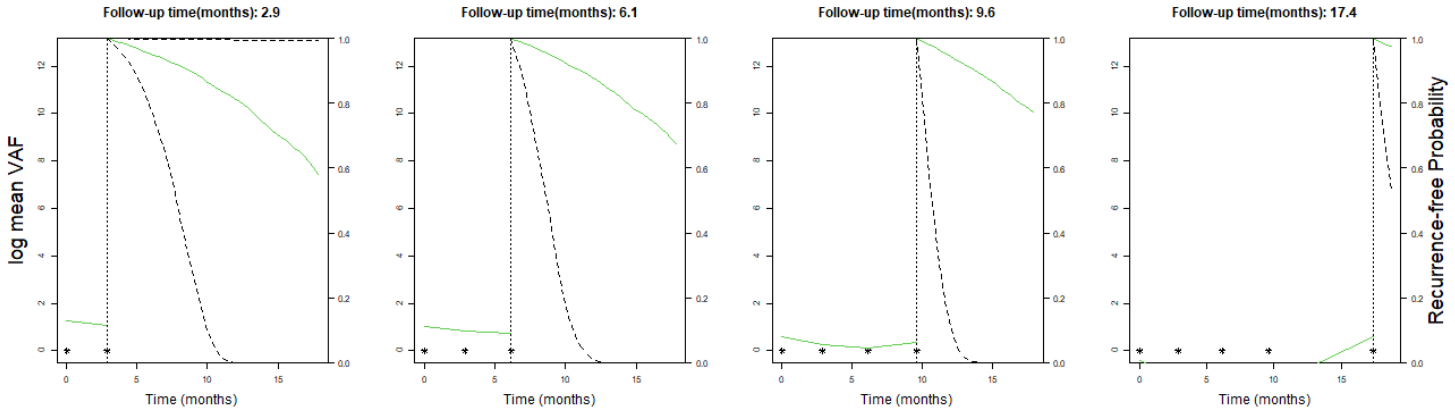

P009, Relaspe-free

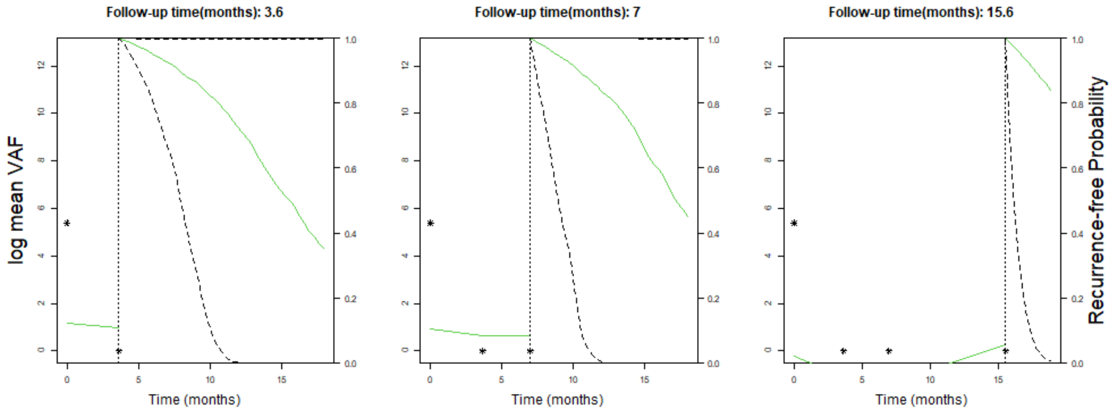

P012, Relaspe-free

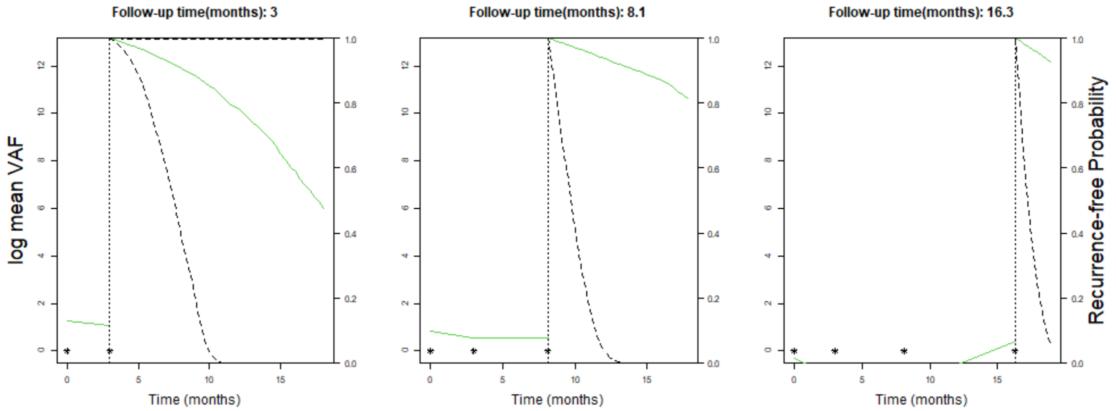

P013, Relapsed

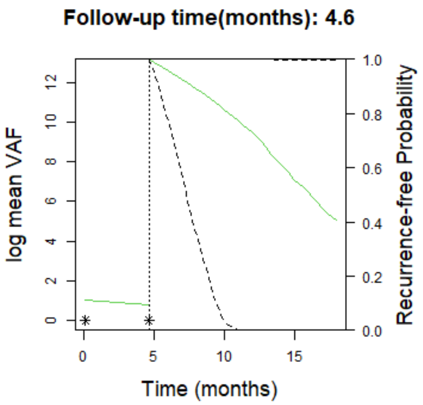

P014, Relaspe-free

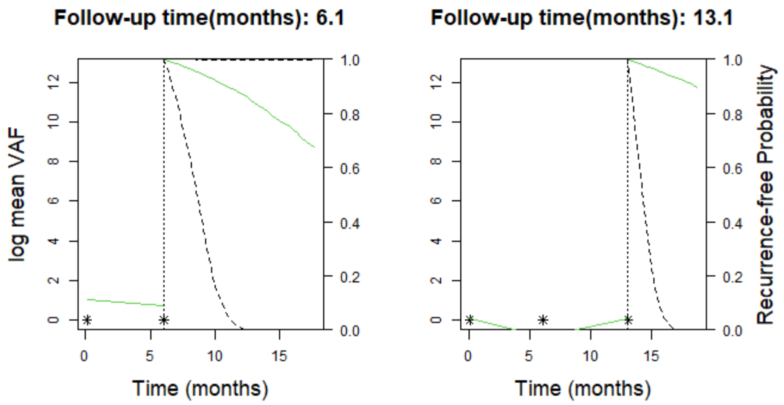

P015, Relaspe-free

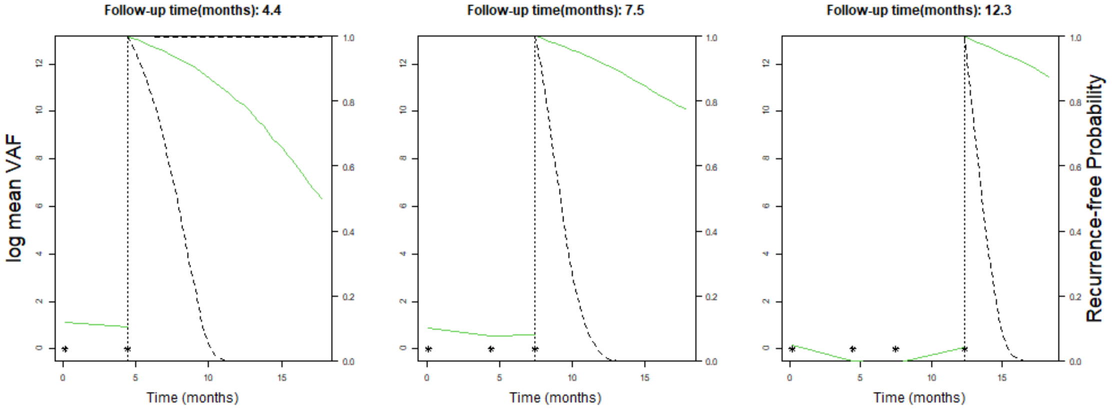

P016, Relapsed

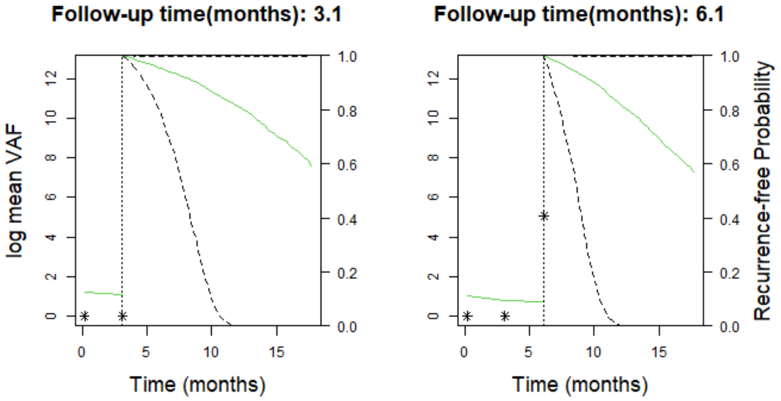

P017, Relapsed

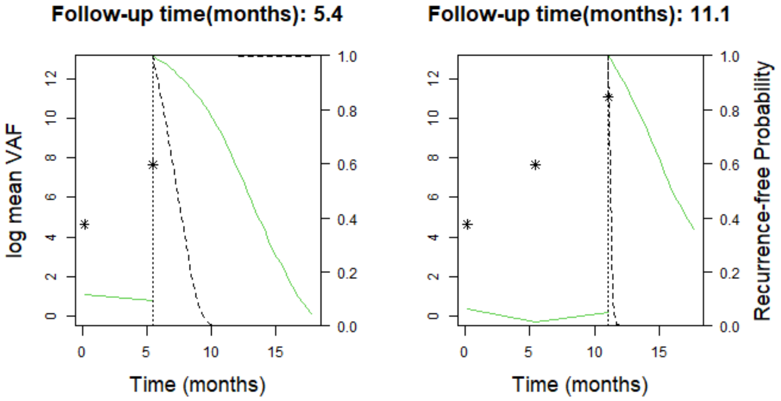

P019, Relapsed

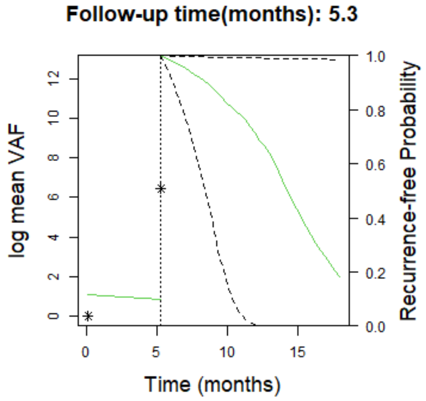

P020, Relaspe-free

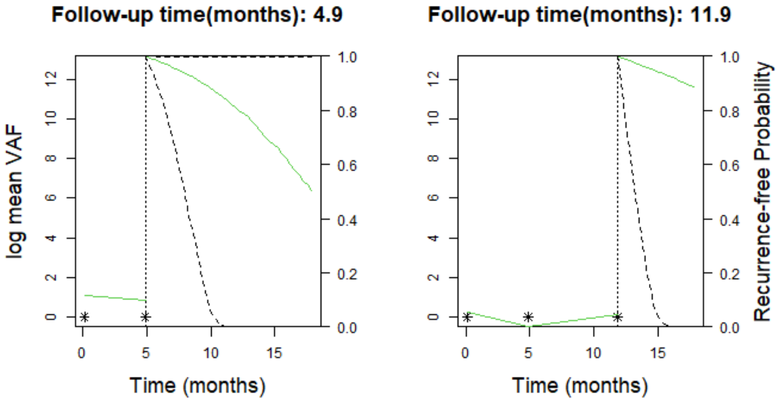

P021, Relapsed

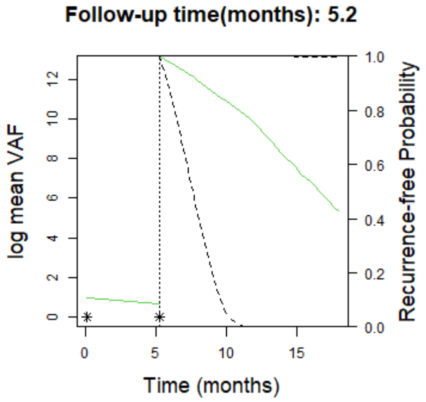

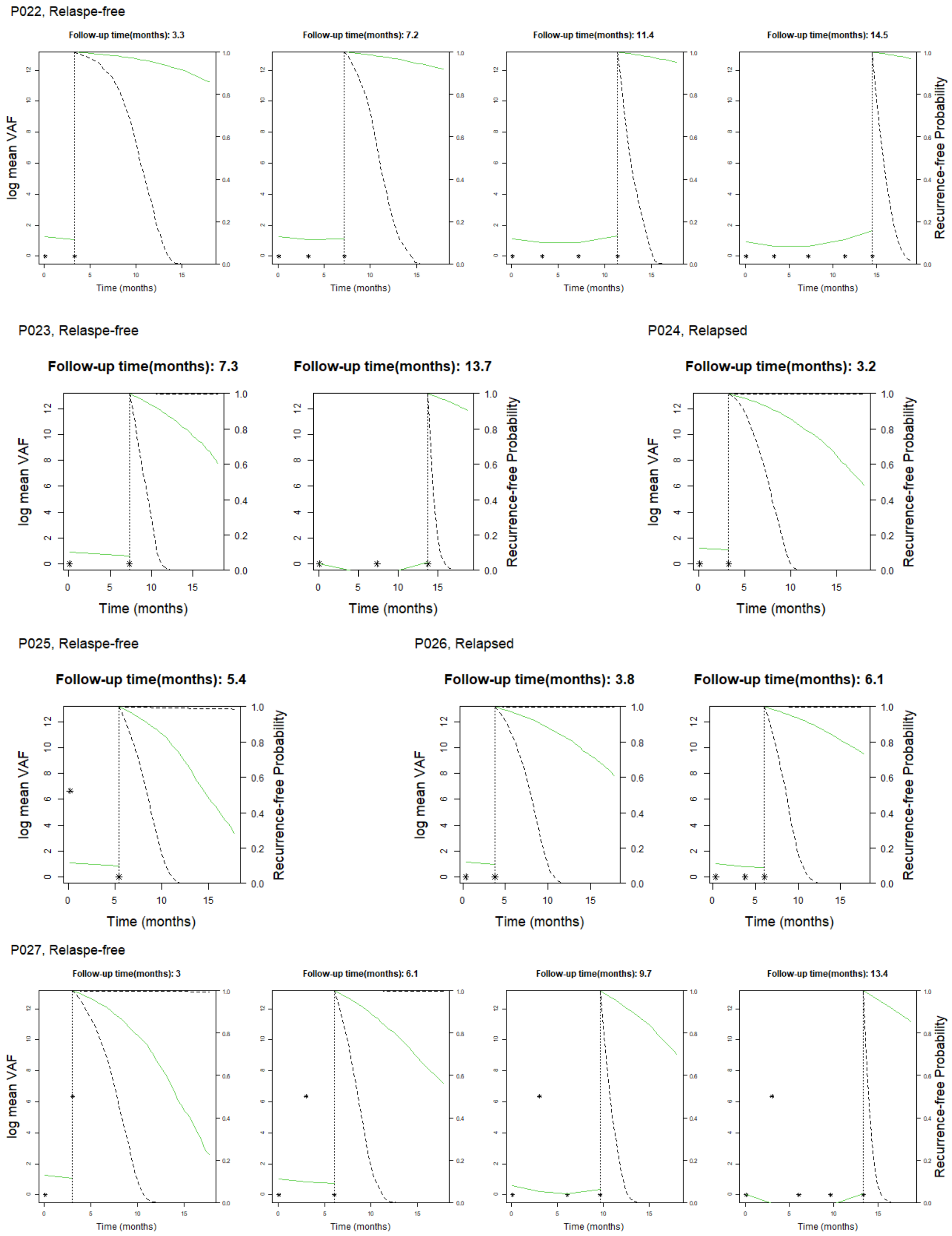

P022, Relaspe-free
Follow-up time(months): 3.3
Follow-up time(months): 7.2
Follow-up time(months): 11.4
Follow-up time(months): 14.5
P023, Relaspe-free
Follow-up time(months): 7.3
Follow-up time(months): 13.7
P024, Relapsed
Follow-up time(months): 3.2
P025, Relaspe-free
Follow-up time(months): 5.4
P026, Relapsed
Follow-up time(months): 3.8
Follow-up time(months): 6.1
P027, Relaspe-free
Follow-up time(months): 3
Follow-up time(months): 6.1
Follow-up time(months): 9.7
Follow-up time(months): 13.4
log mean VAF
Recurrence-free Probability
Time (months)

P028, Relapsed

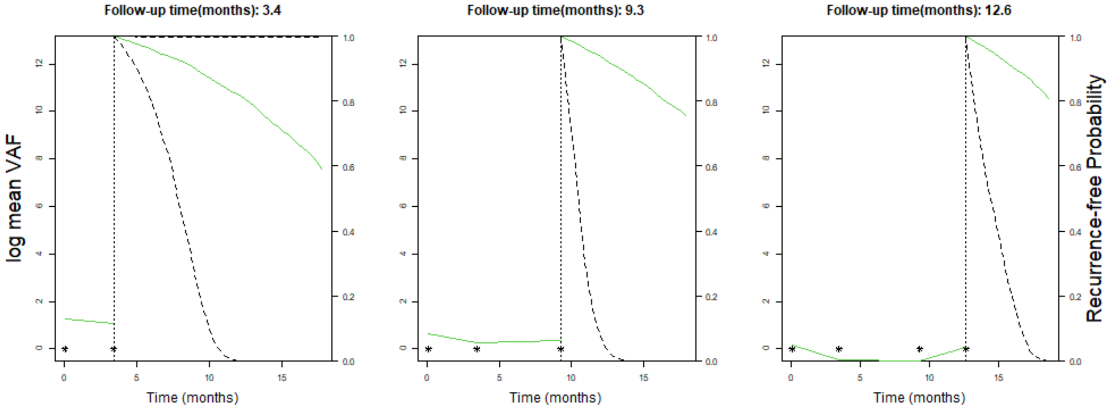

P029, Relaspe-free

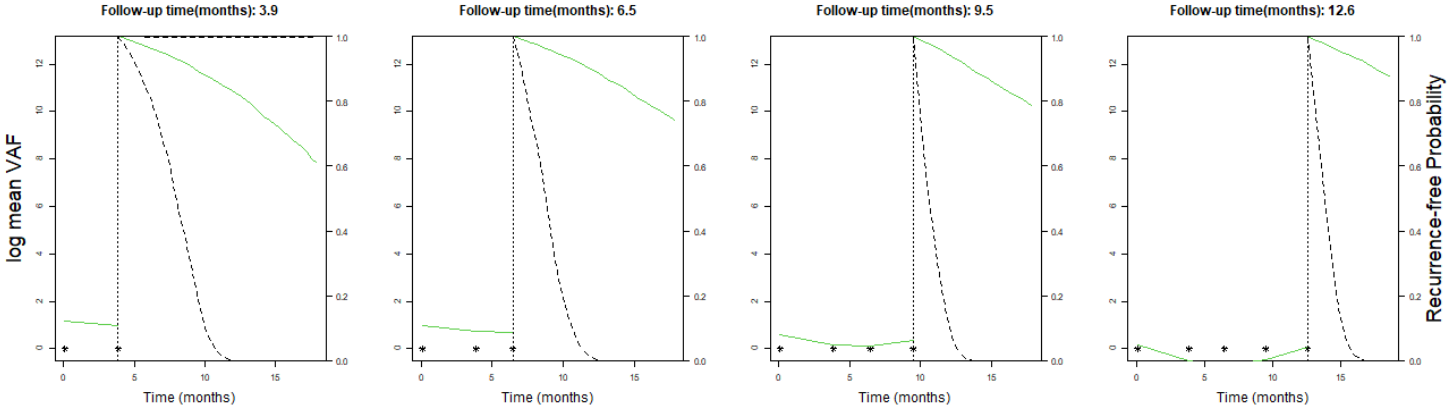

P032, Relaspe-free

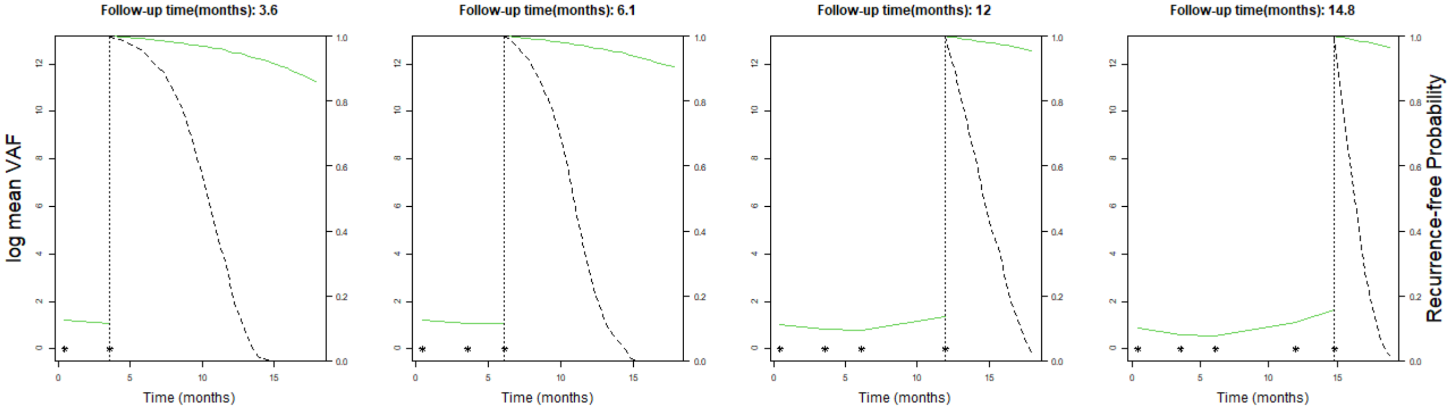

P030, Relapsed

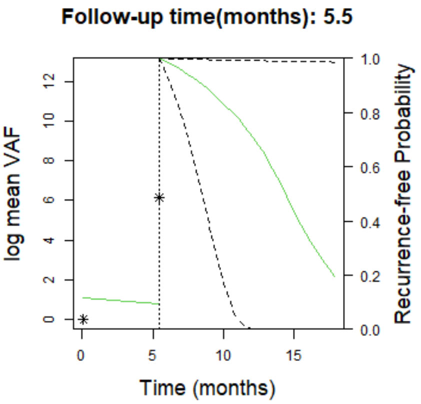

P033, Relaspe-free

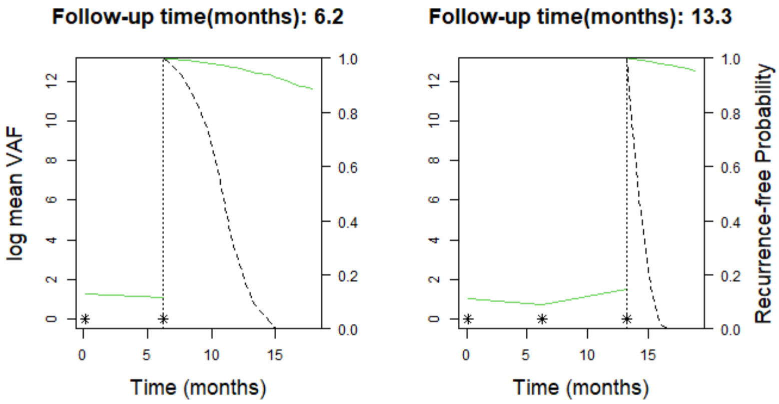

P034, Relapsed

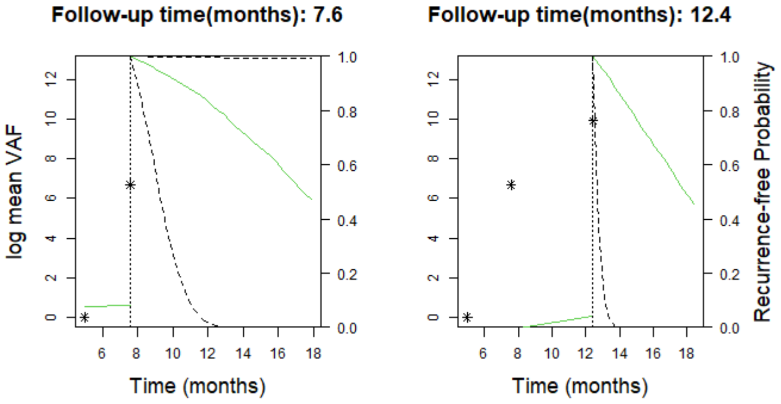

P035, Relaspe-free

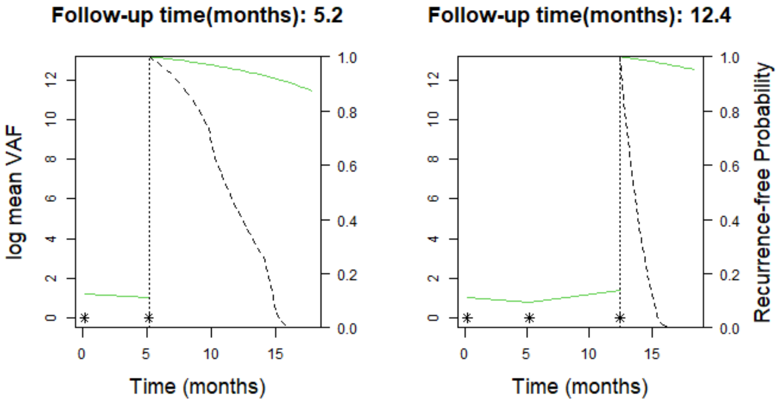

P037, Relapsed

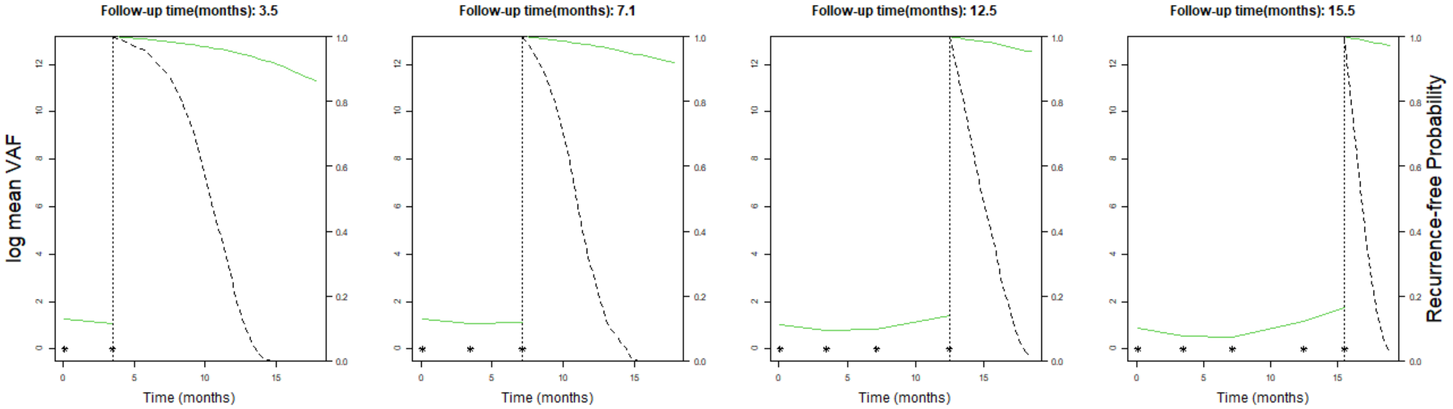

P039, Relaspe-free

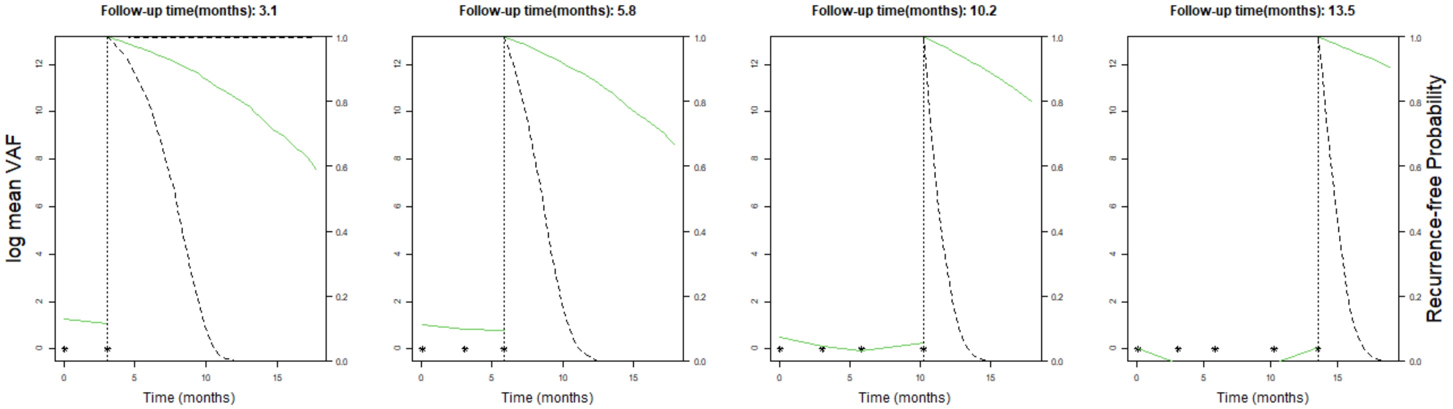

P040, Relaspe-free

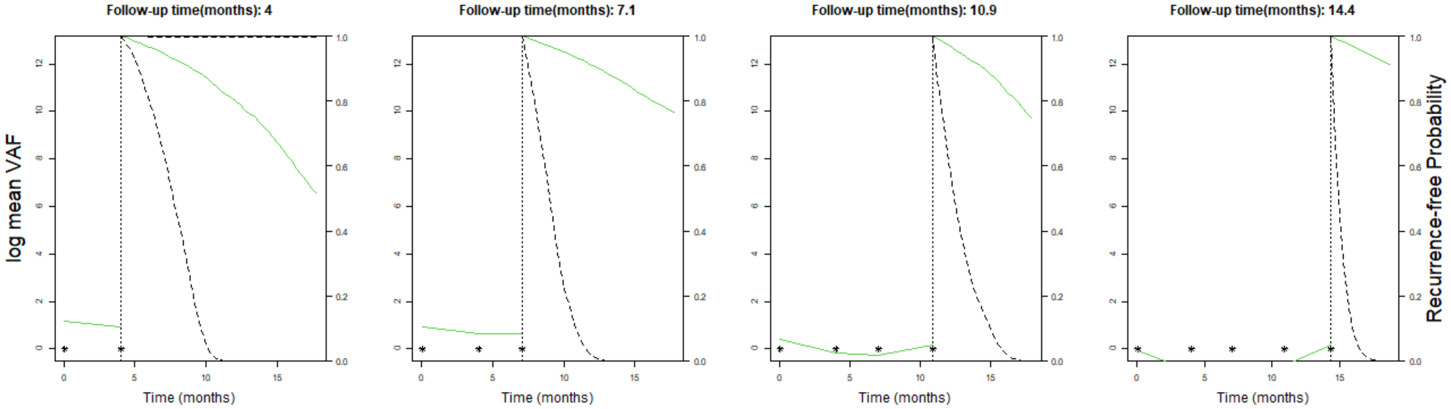

P041, Relaspe-free

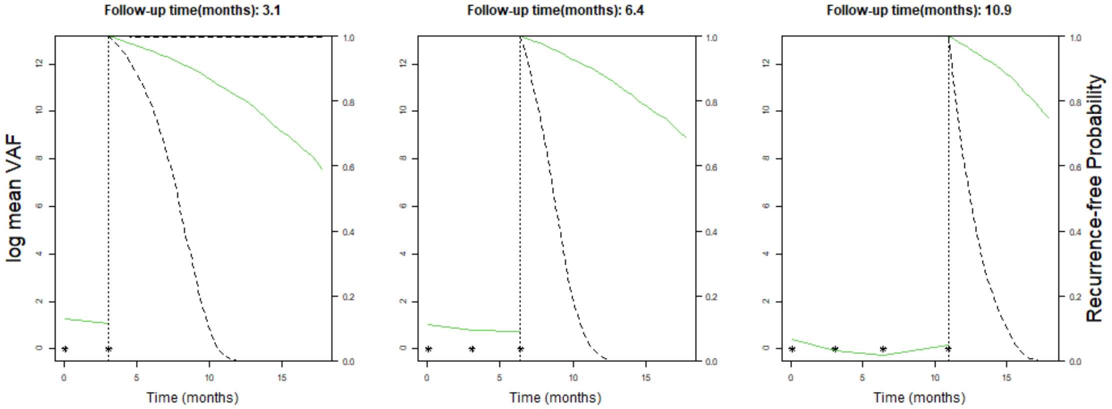

P042, Relaspe-free

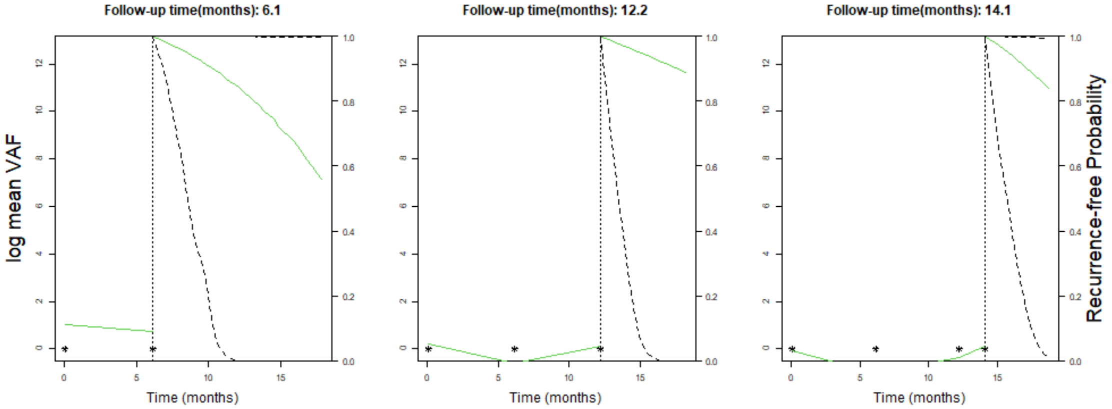

P043, Relaspe-free

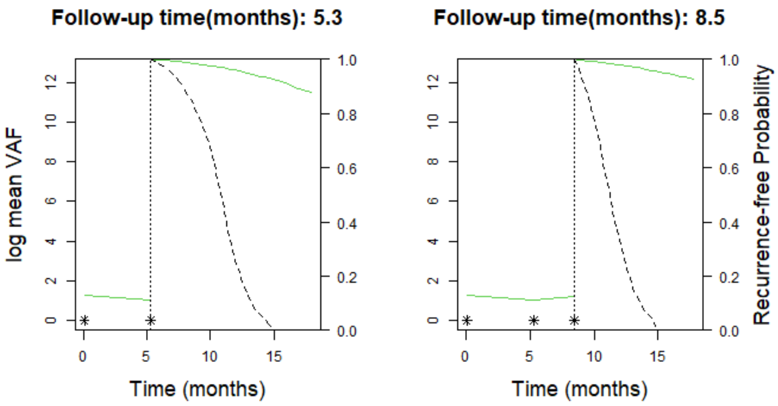

P002, Relaspe-free

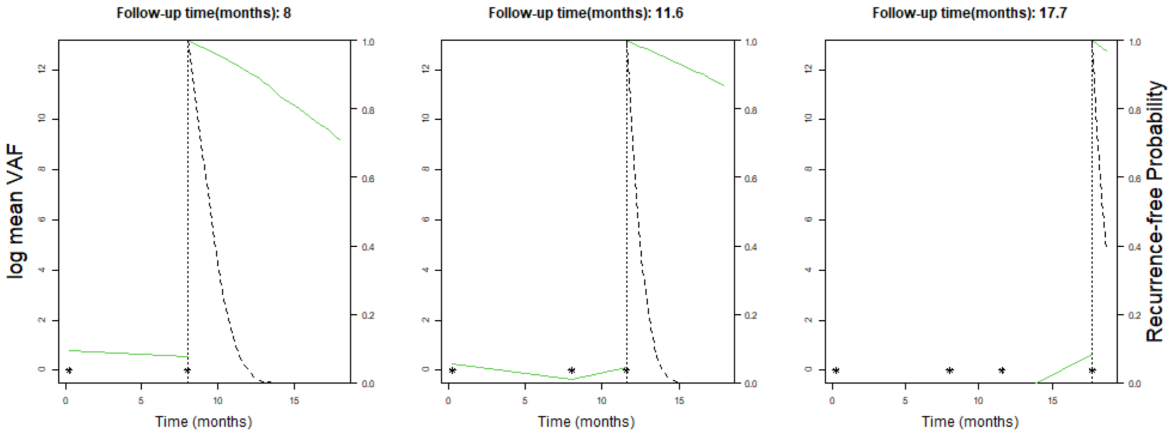

P003, Relaspe-free

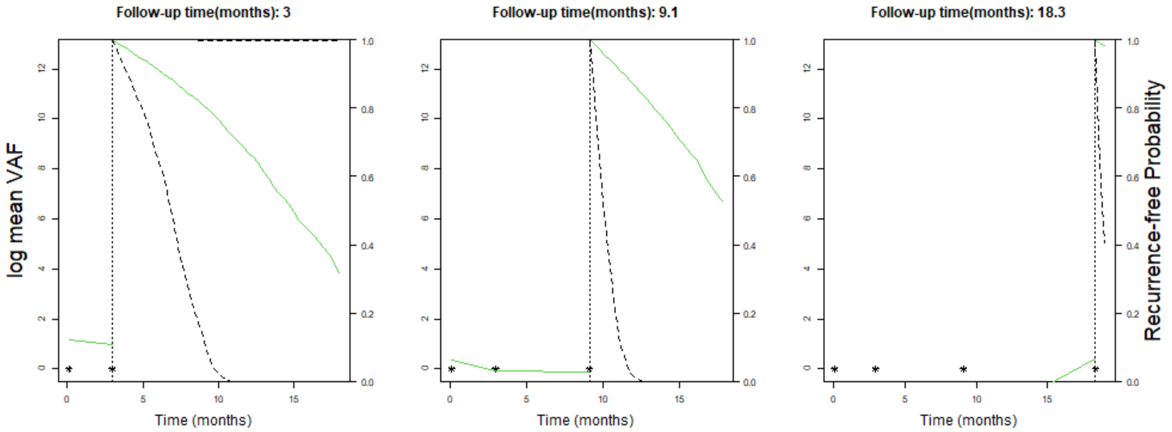

P004, Relaspe-free

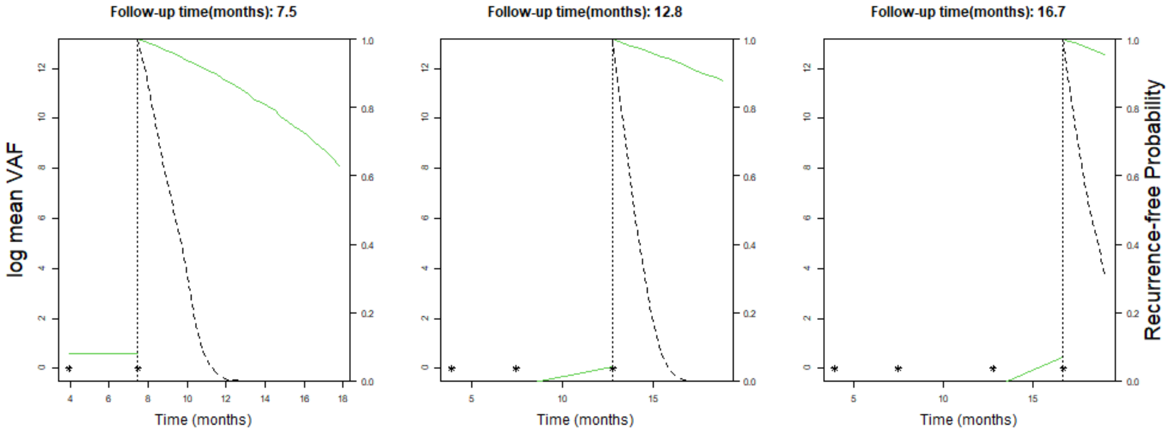

P005, Relapsed

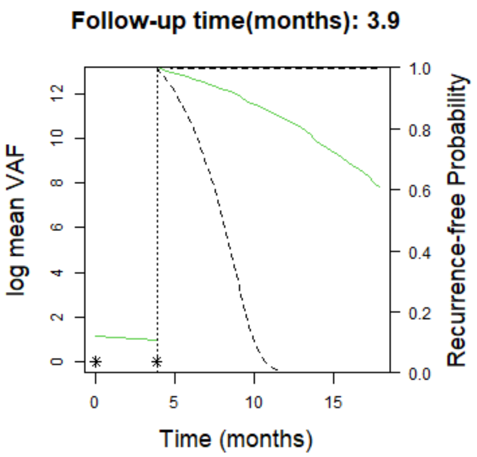

P006, Relapsed

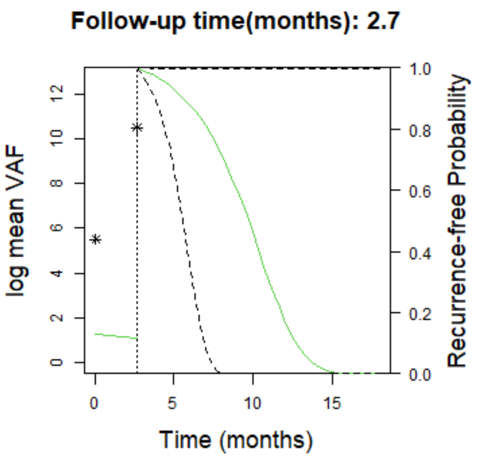

P008, Relaspe-free

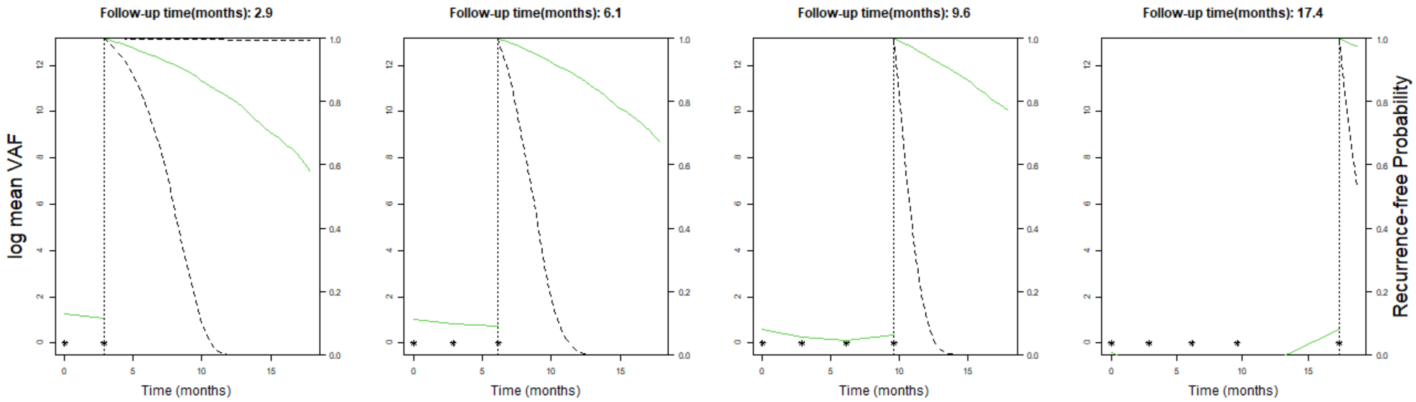

P009, Relapse-free

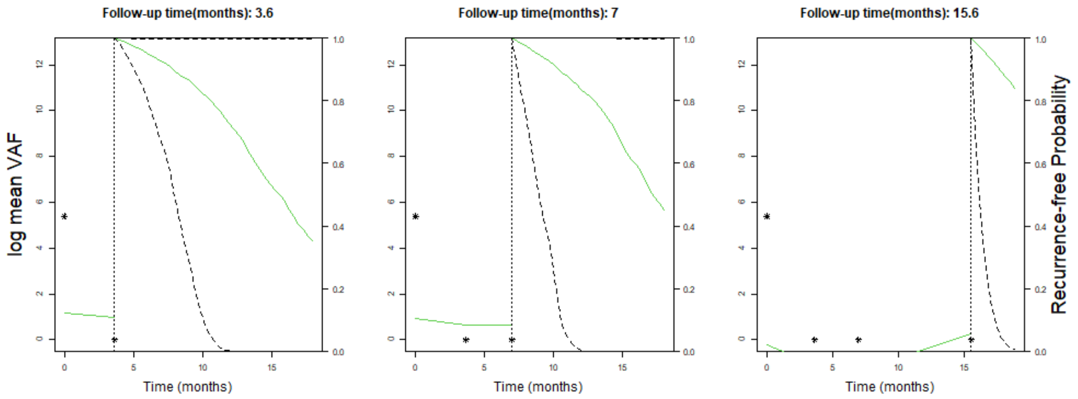

P012, Relaspe-free

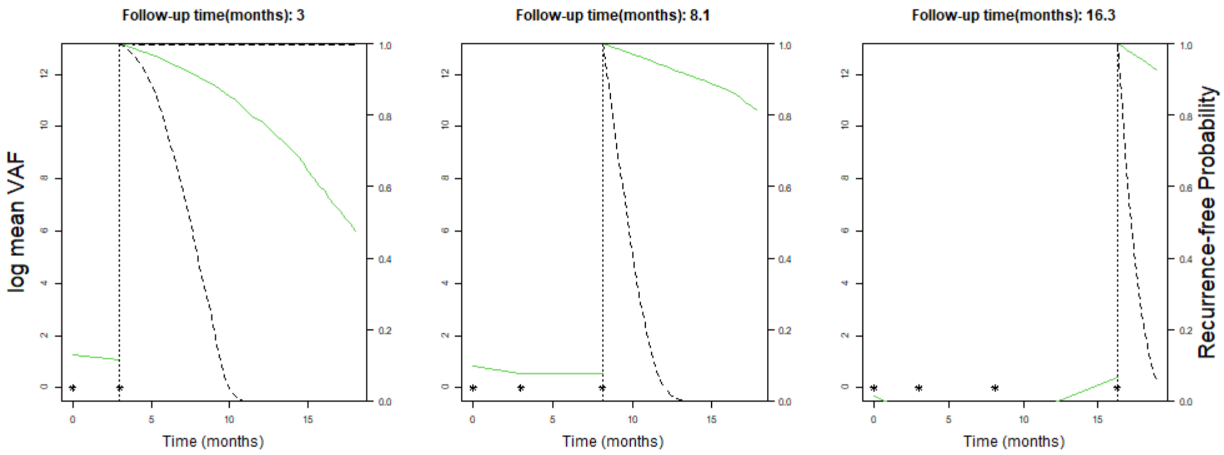

P013, Relapsed

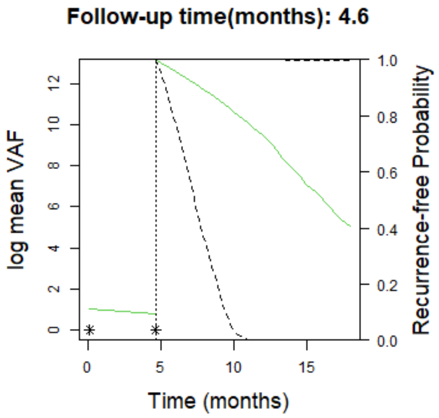

P014, Relaspe-free

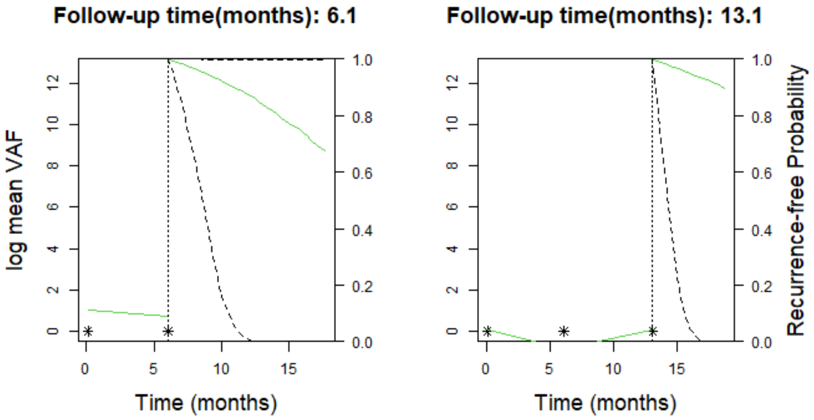

P015, Relaspe-free

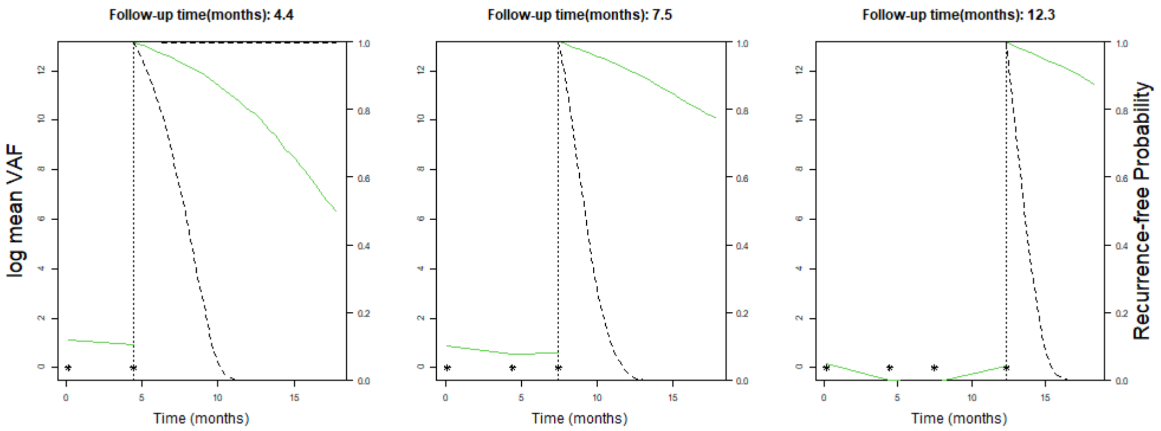

P016, Relapsed

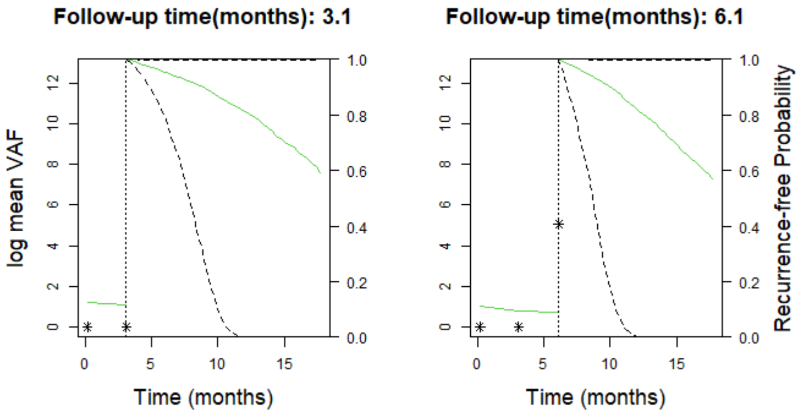

P017, Relapsed

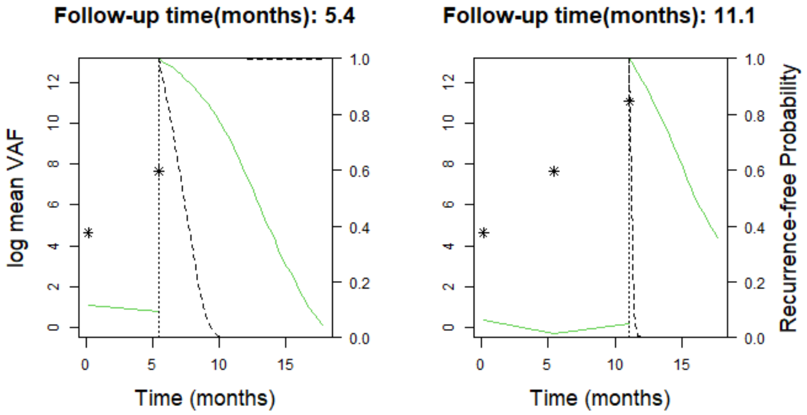

P019, Relapsed

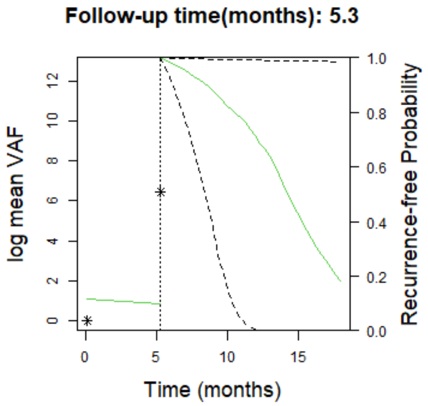

P020, Relaspe-free

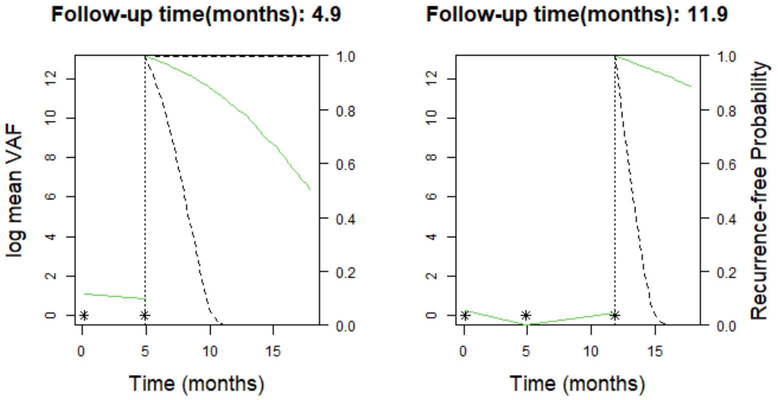

P021, Relapsed

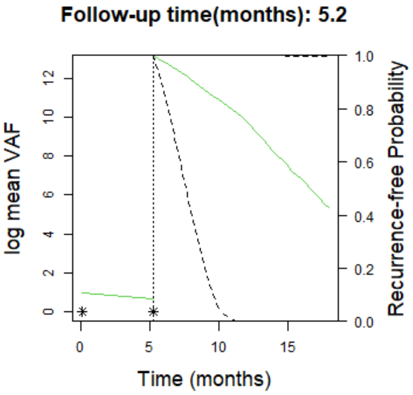

P022, Relaspe-free

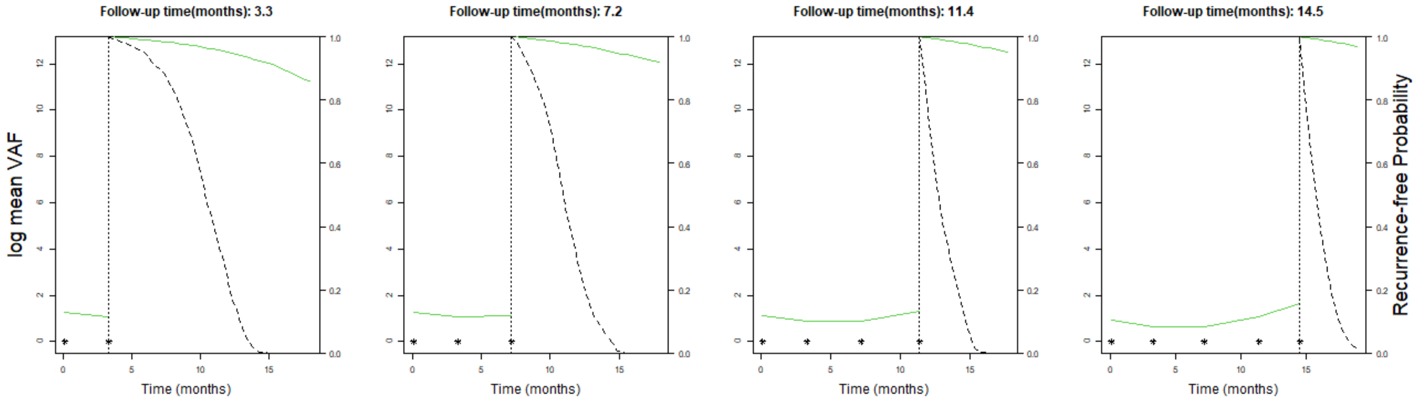

P023, Relaspe-free

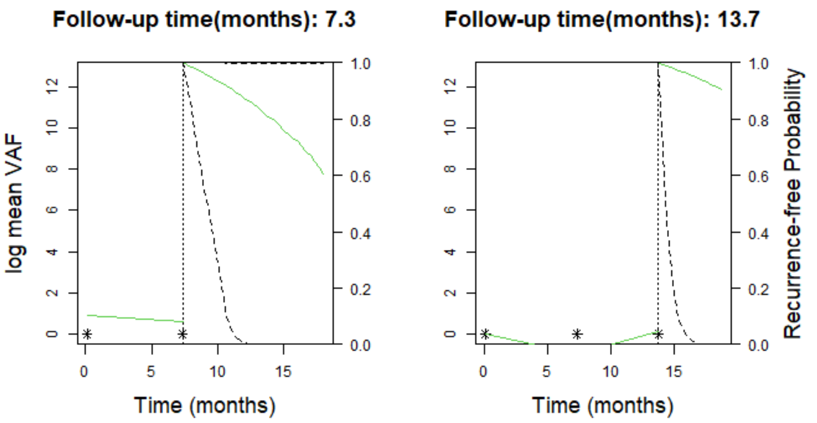

P024, Relapsed

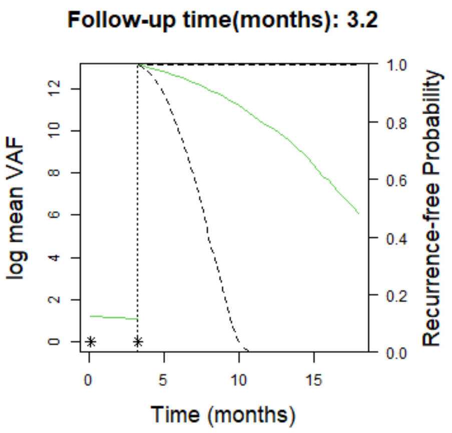

P025, Relaspe-free

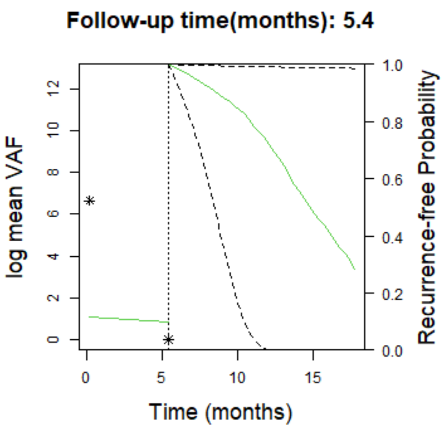

P026, Relapsed

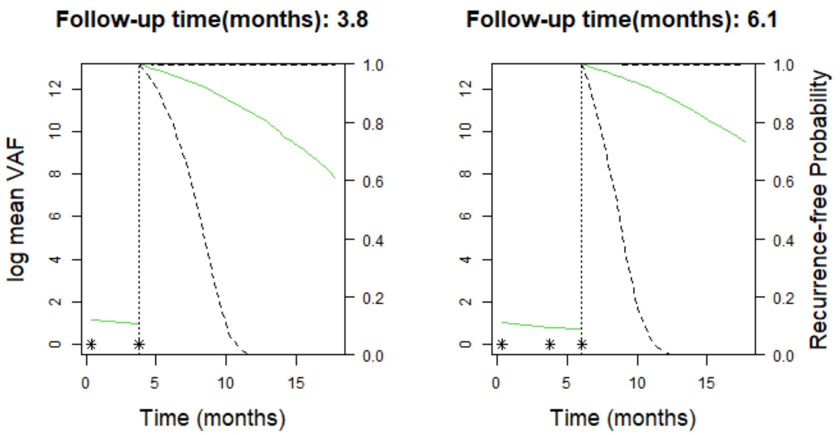

P027, Relaspe-free

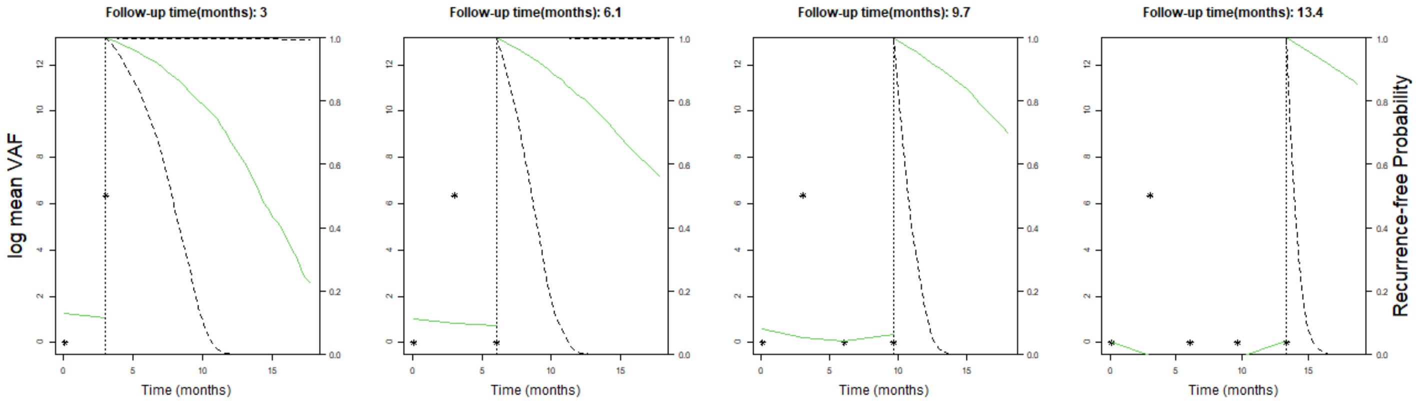

P028, Relapsed

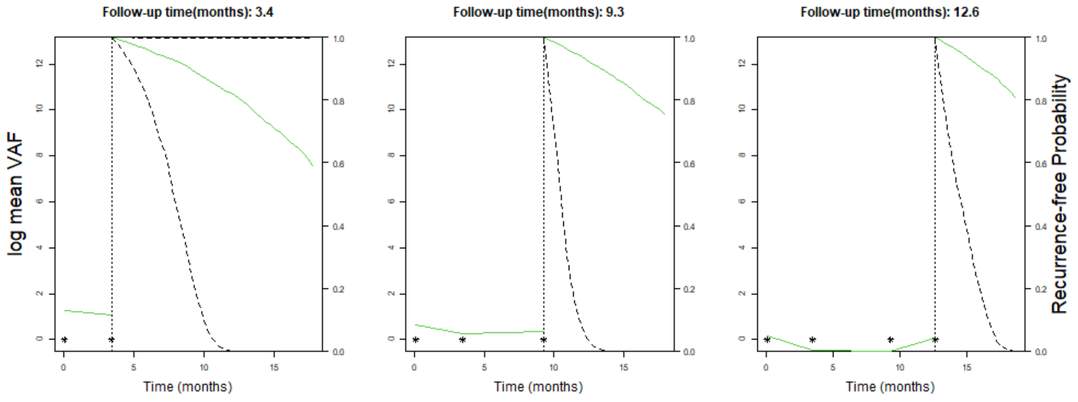

P029, Relaspe-free

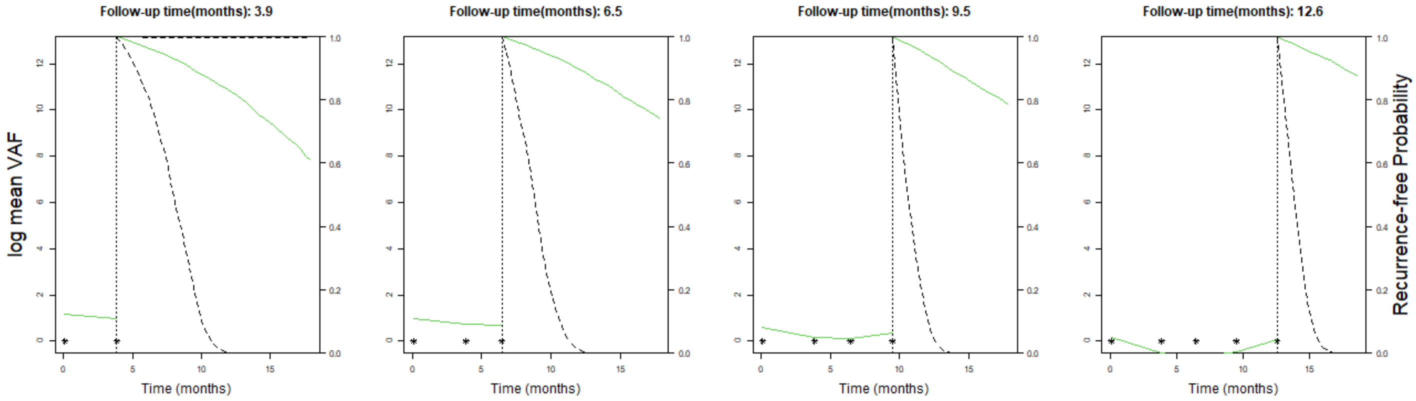

P030, Relapsed

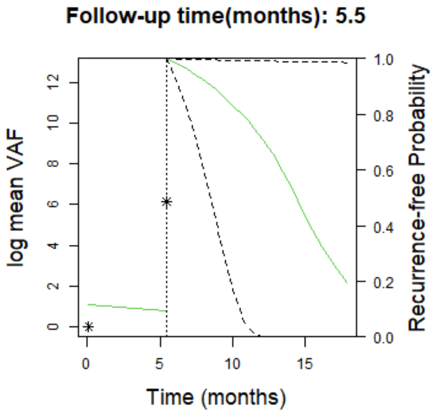

P033, Relaspe-free

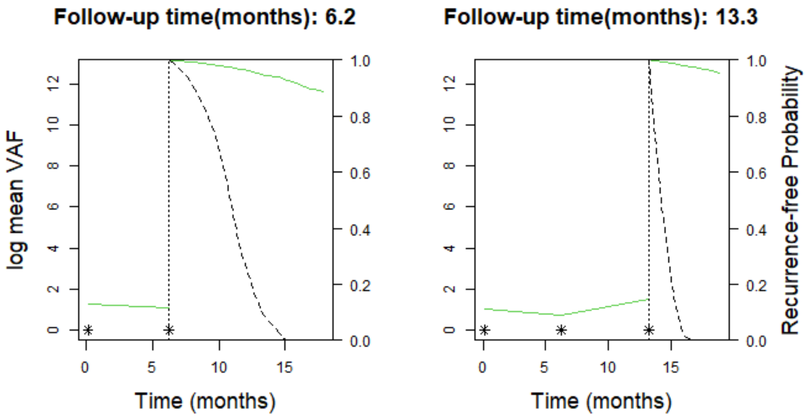

P032, Relaspe-free

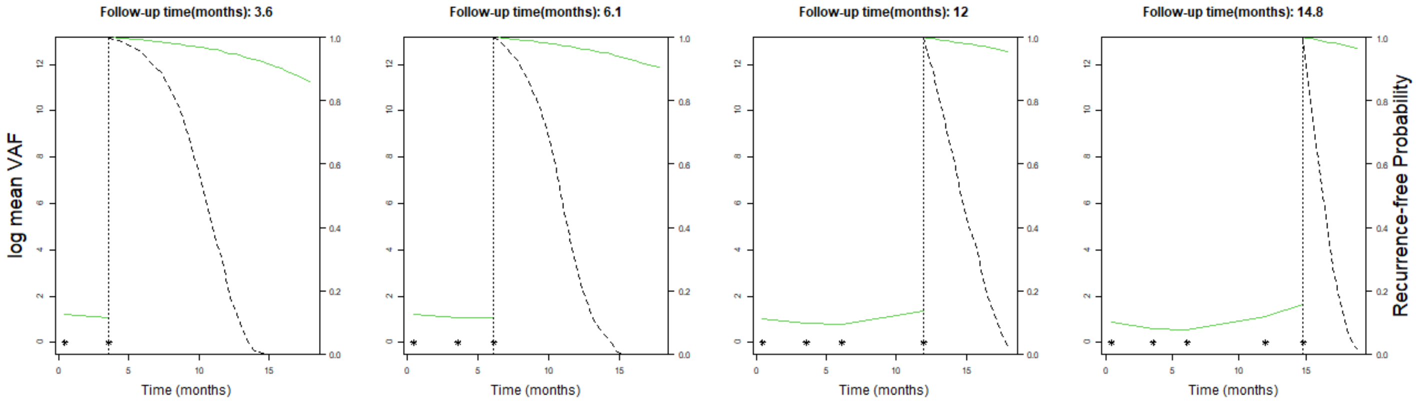

P034, Relapsed

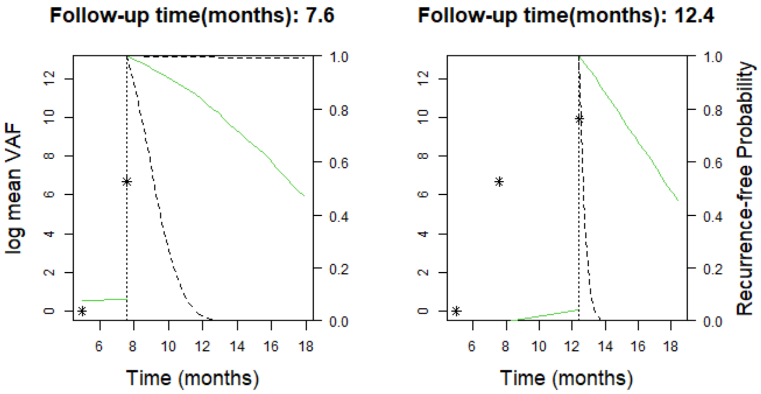

P035, Relaspe-free

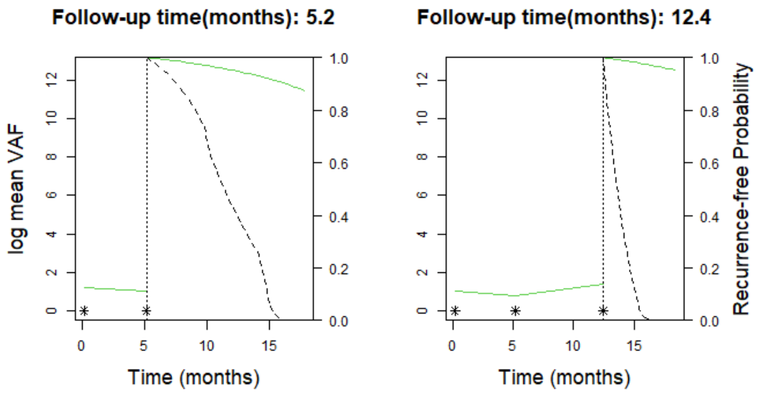

P037, Relapsed

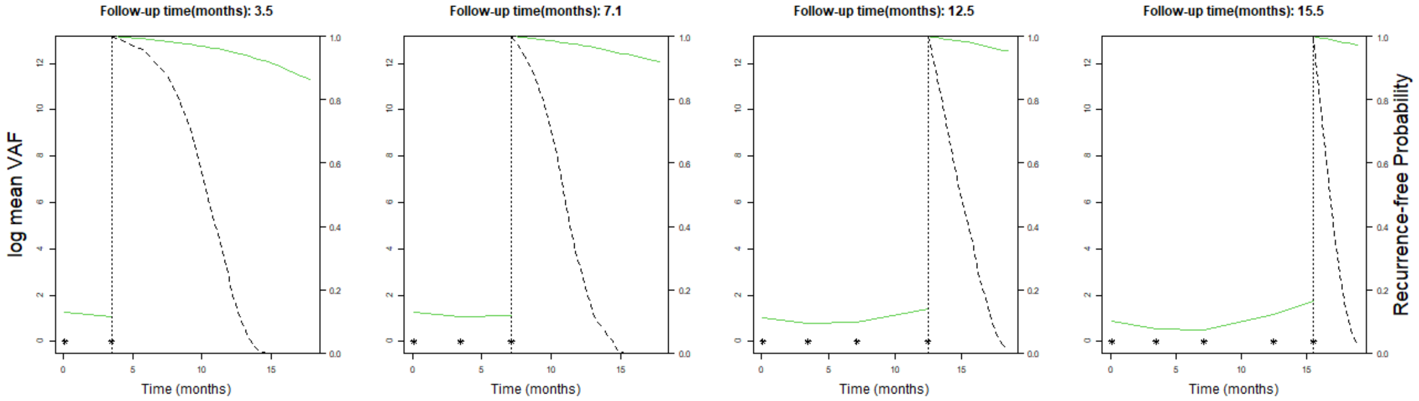

P039, Relaspe-free

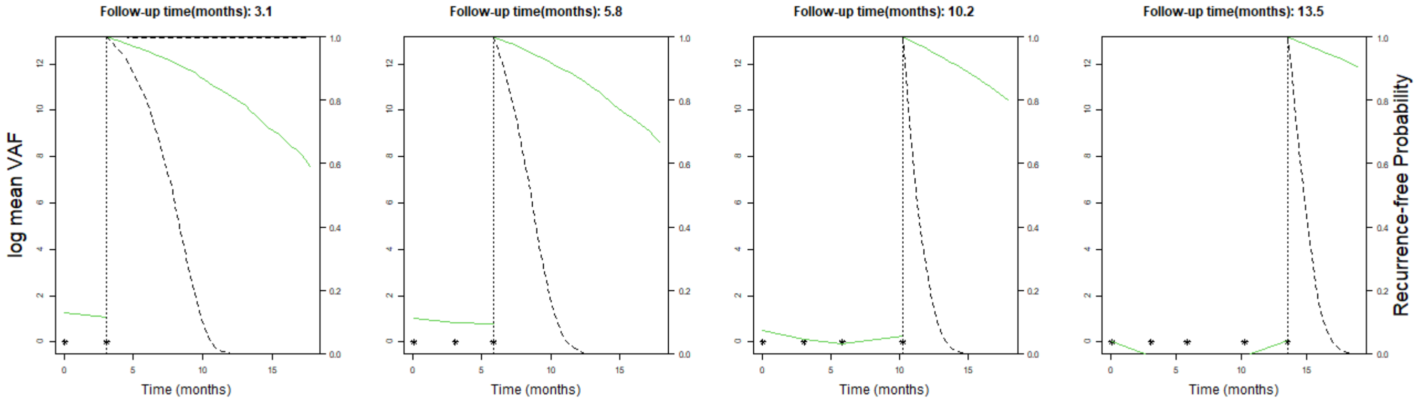

P040, Relaspe-free

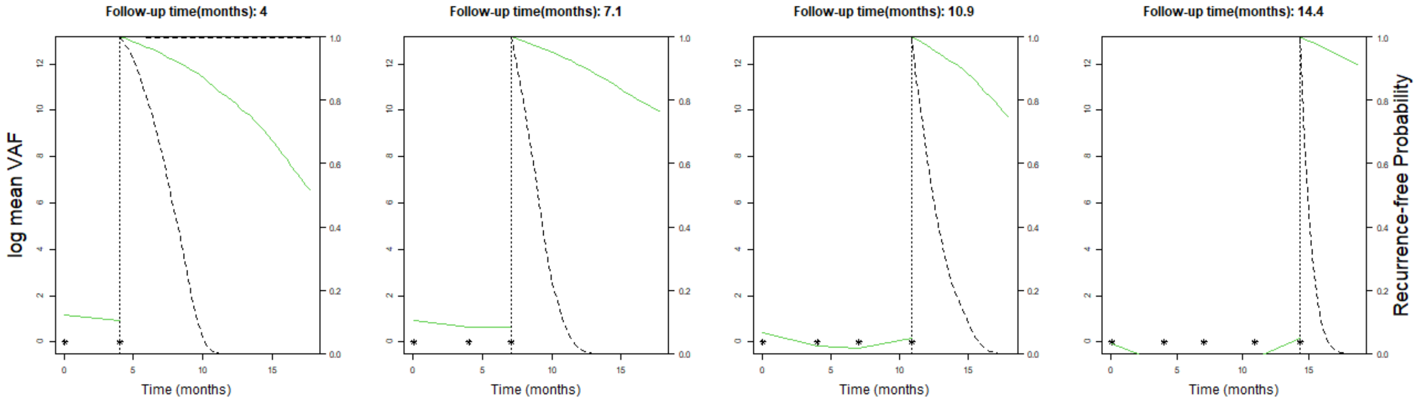

P041, Relaspe-free

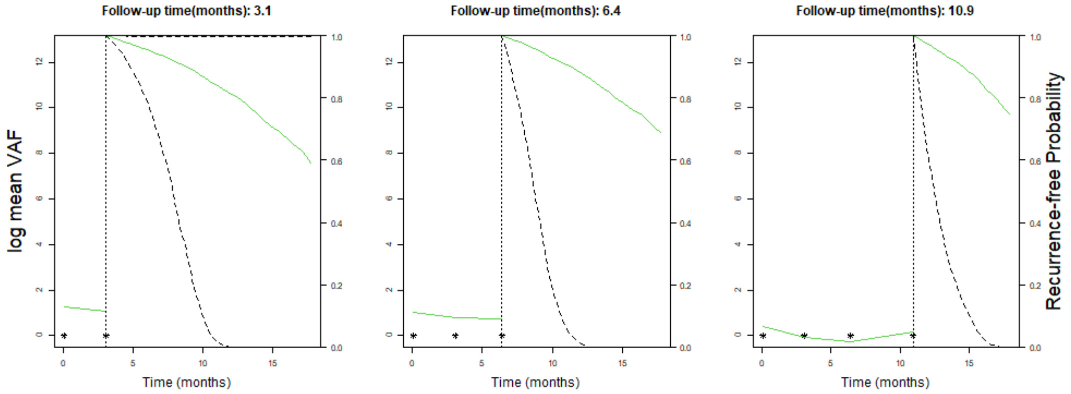

P042, Relaspe-free

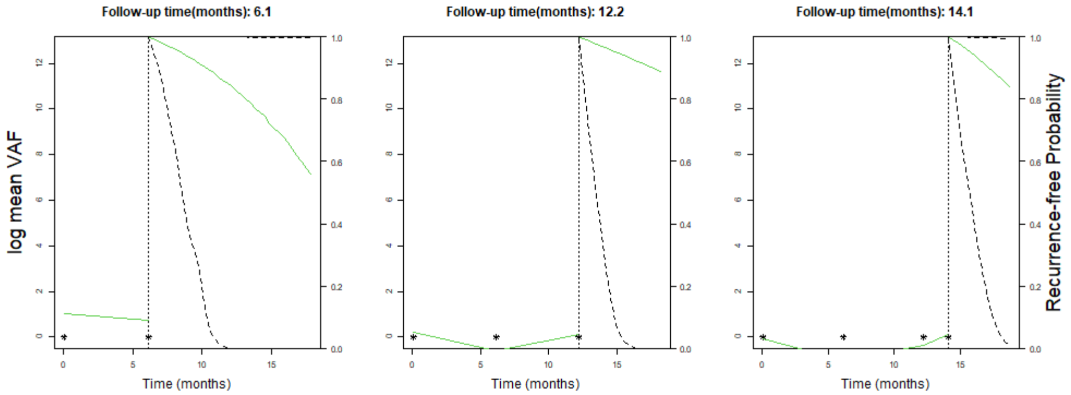

P043, Relaspe-free

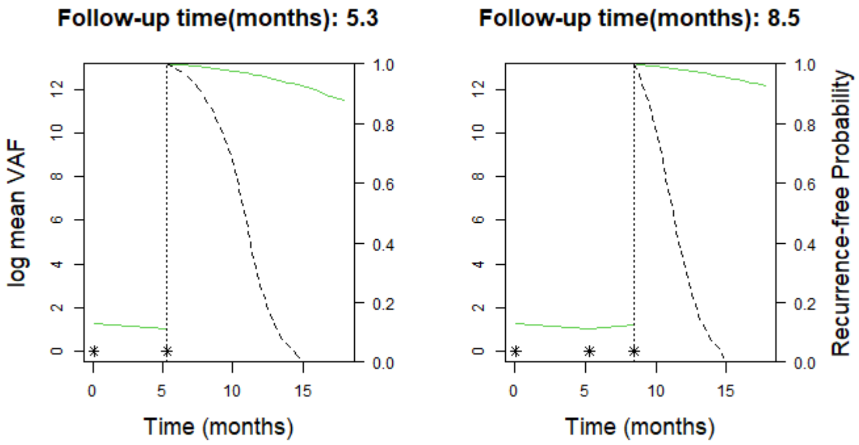

P045, Relaspe-free

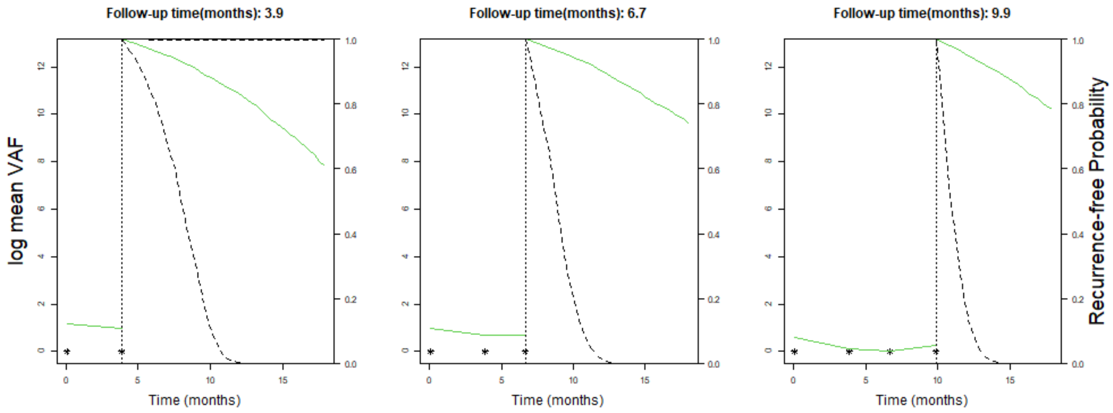

P047, Relaspe-free

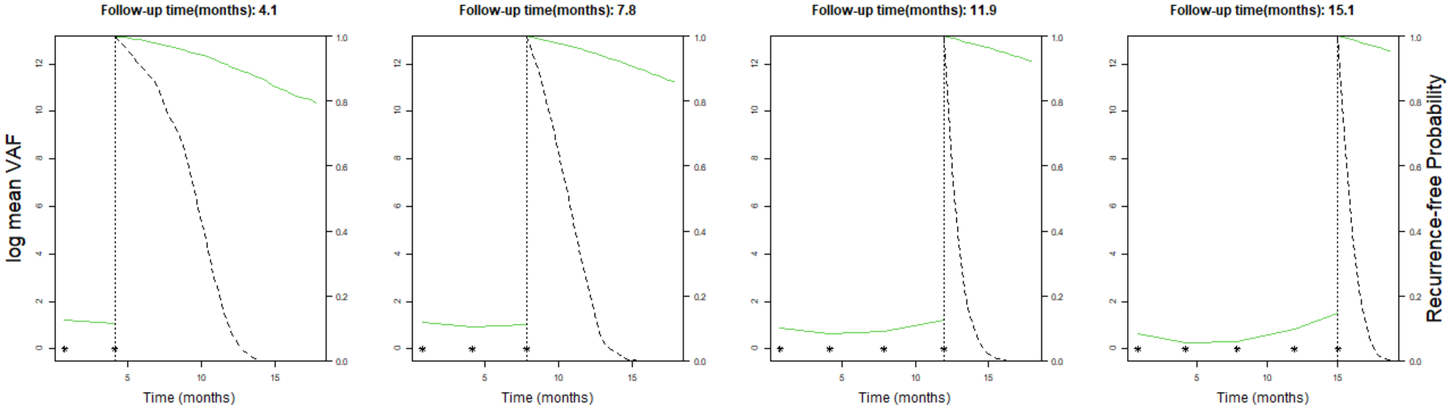

P048, Relaspe-free

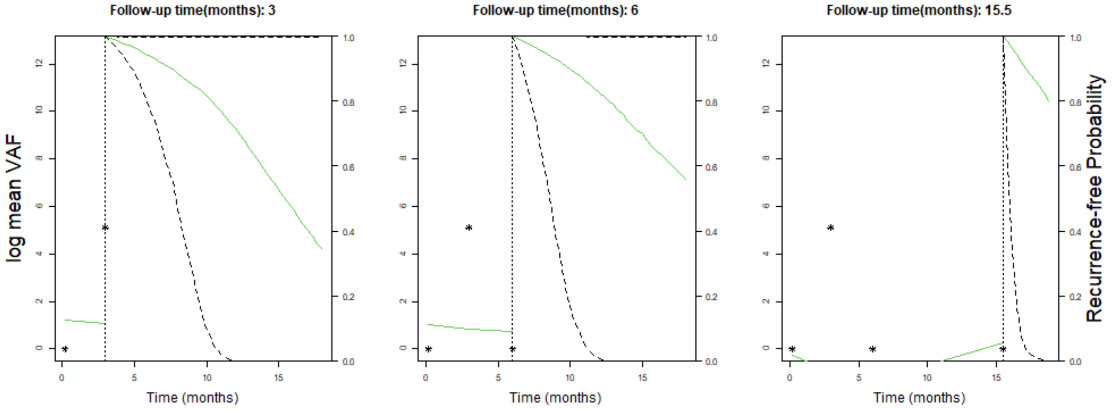

P049, Relaspe-free

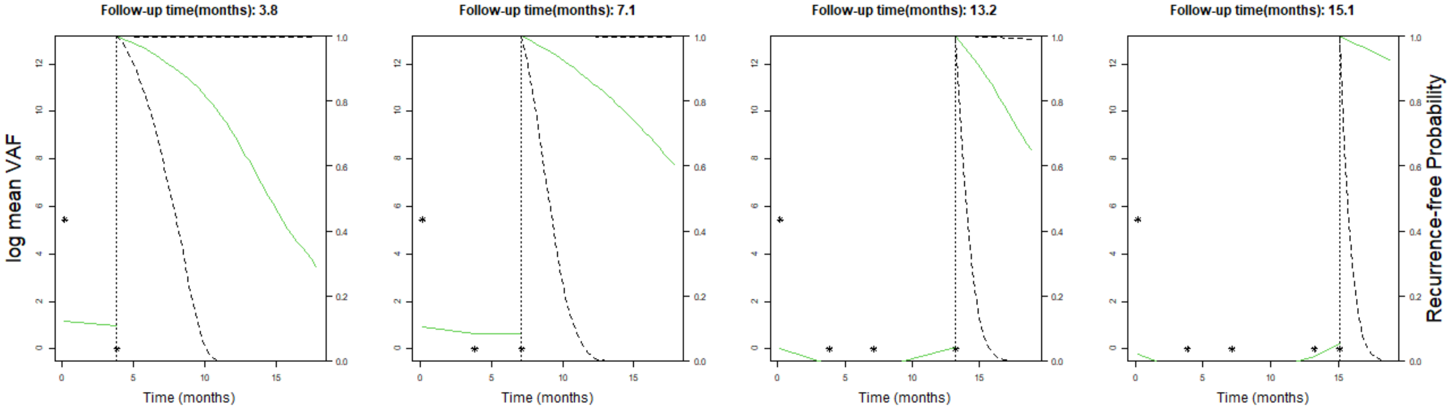

P050, Relaspe-free

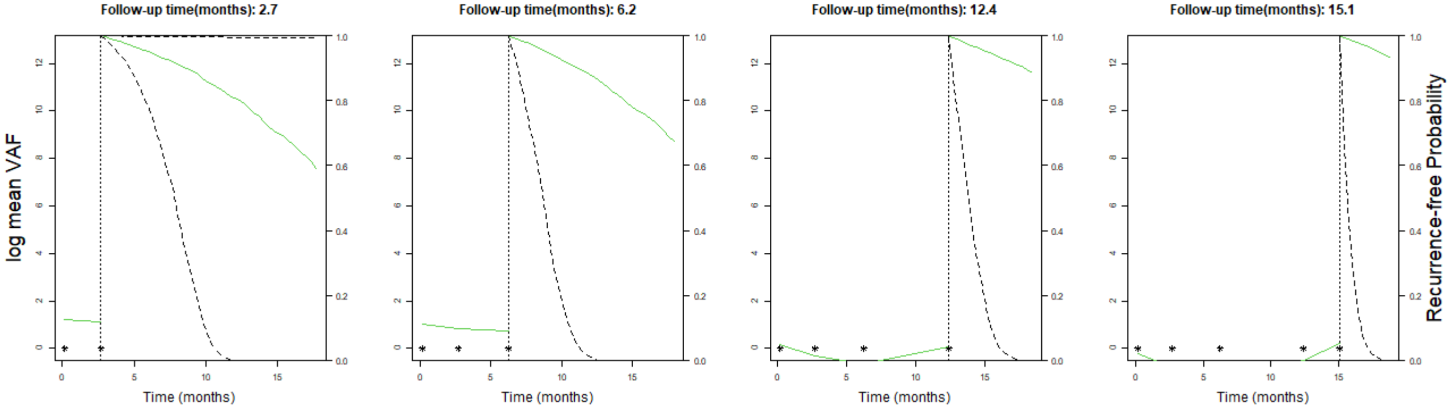

P053, Relaspe-free

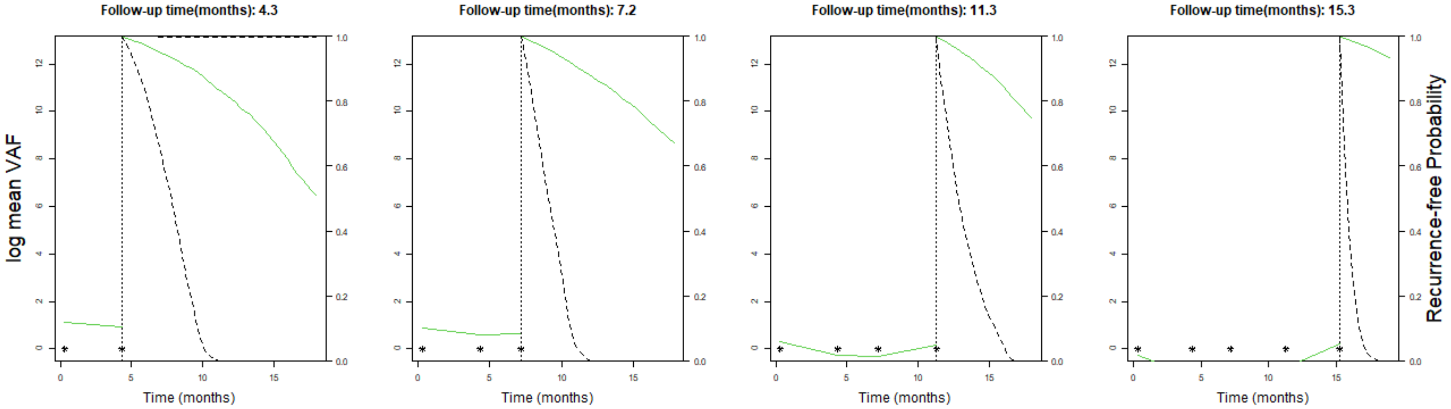

P054, Relapsed

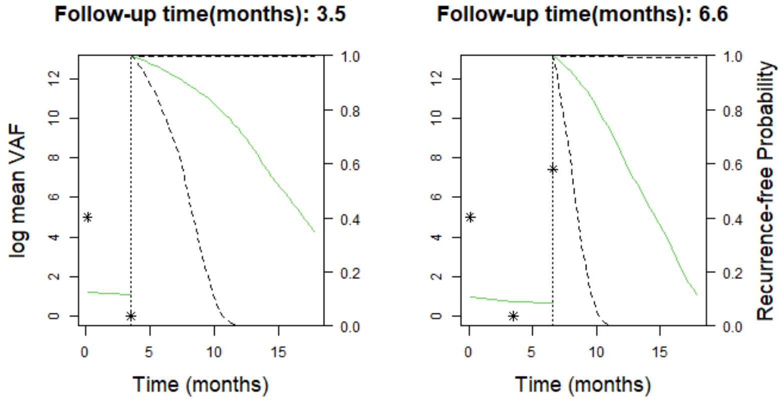

P051, Relapsed

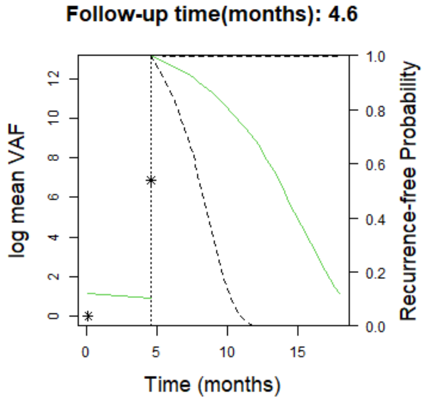

P055, Relaspe-free

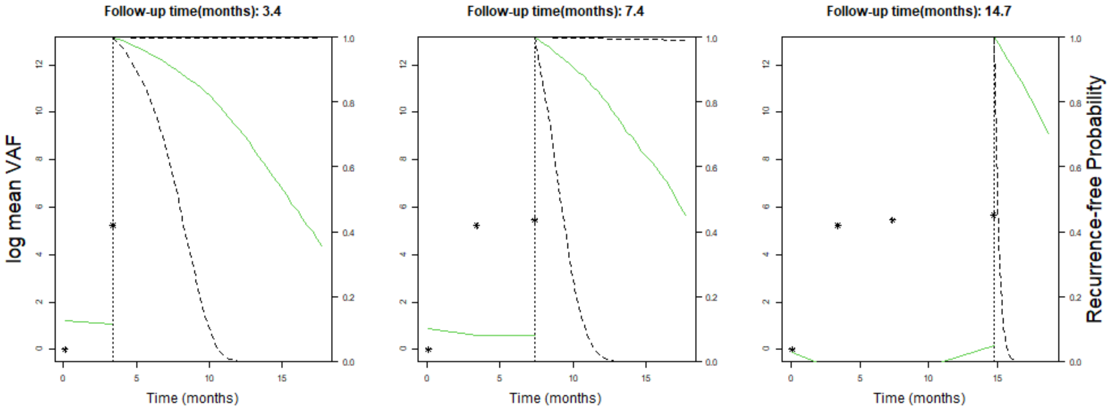

P056, Relaspe-free

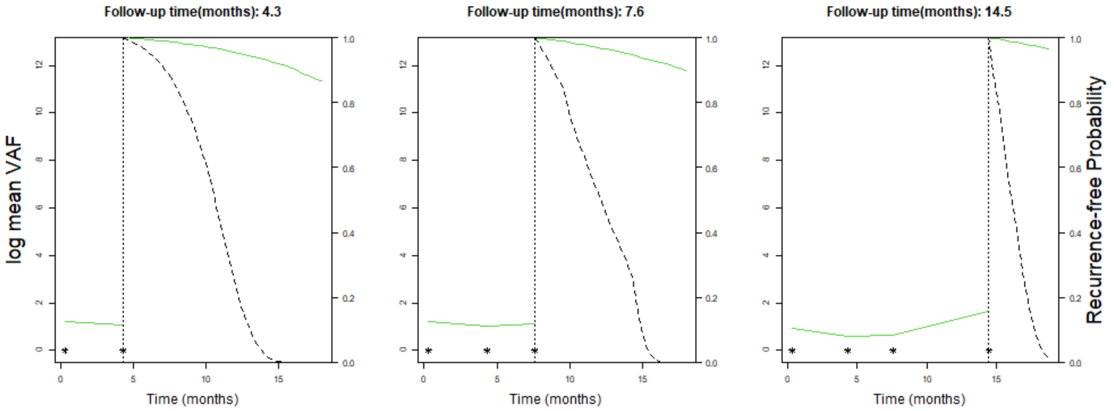

P057, Relapsed

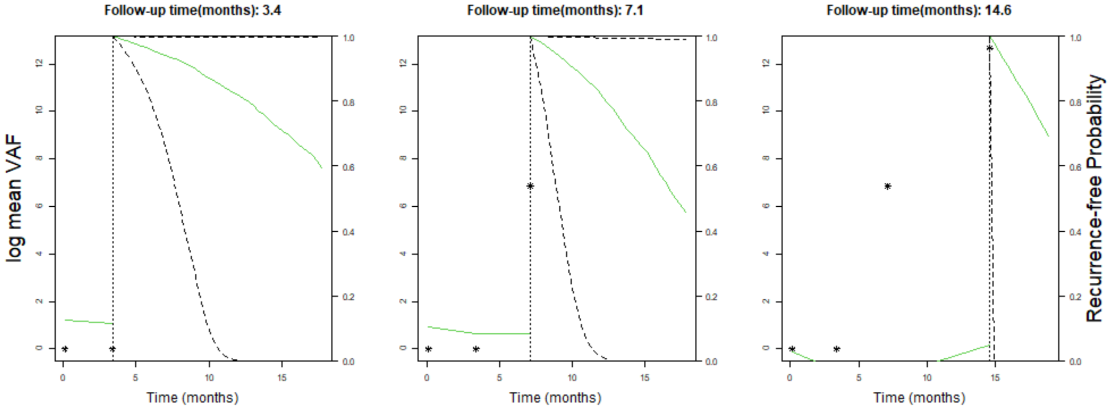

P058, Relaspe-free

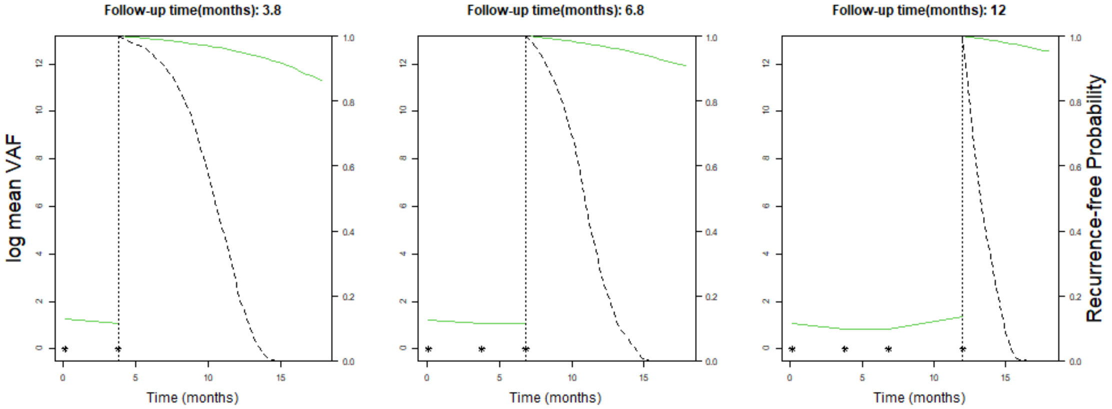

P061, Relaspe-free

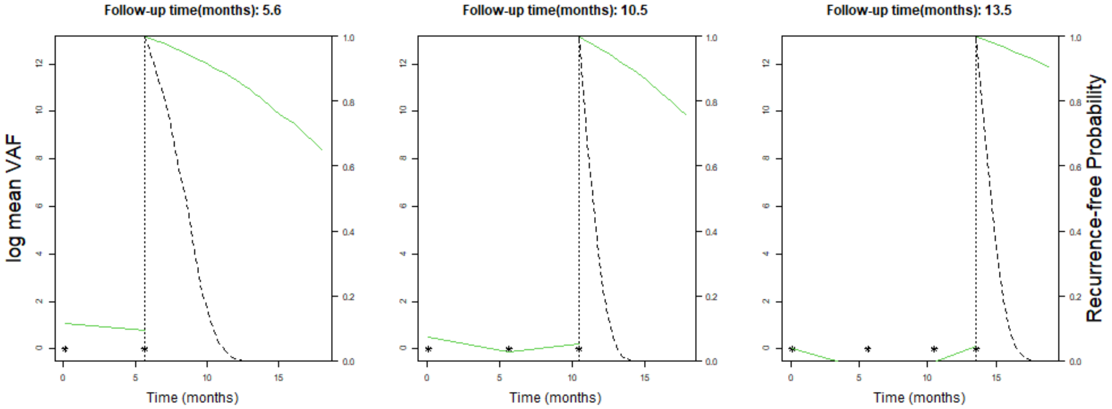

P063, Relaspe-free

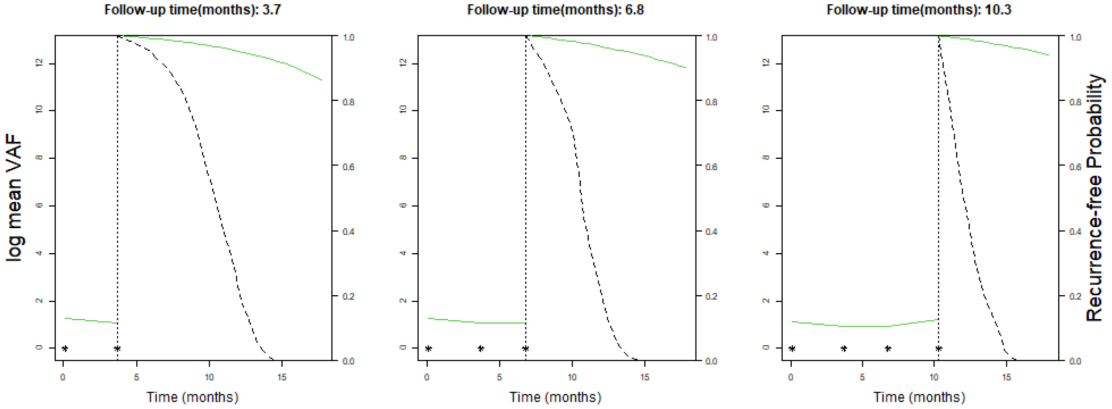

P064, Relaspe-free

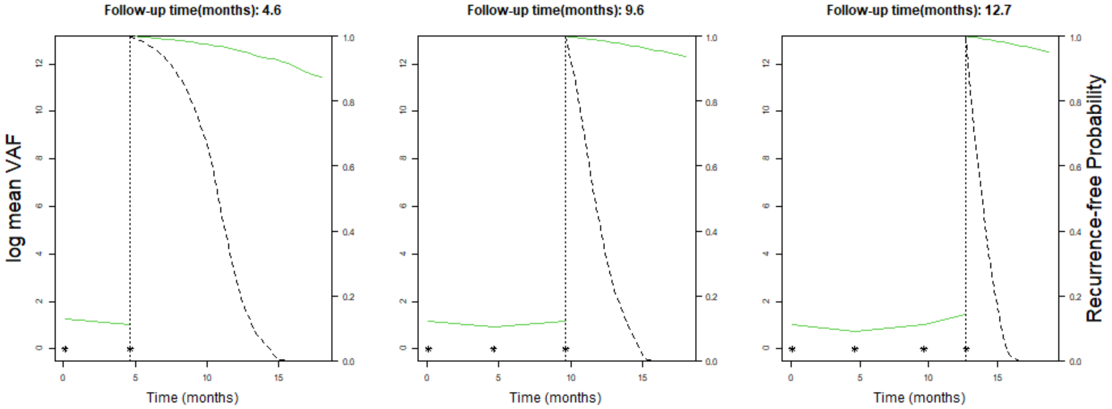

P060, Relapsed

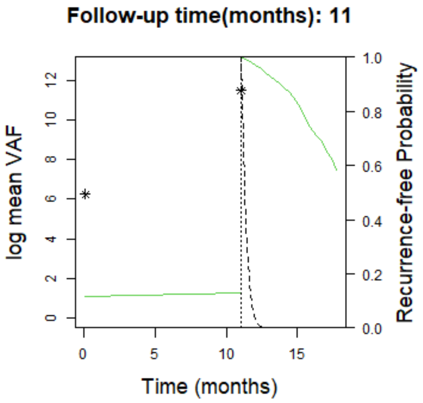

P065, Relaspe-free

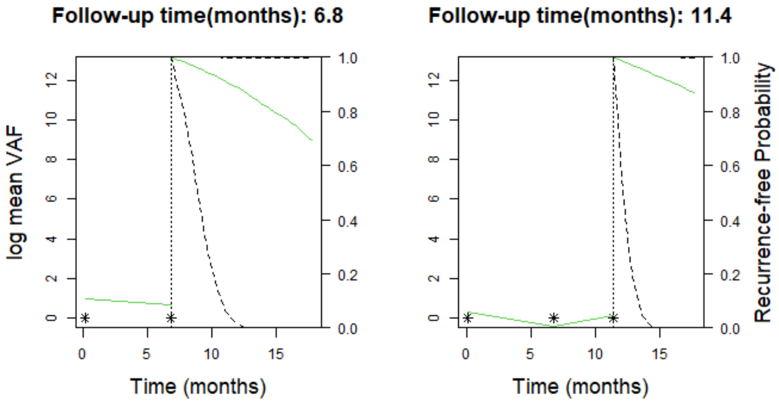

P067, Relaspe-free

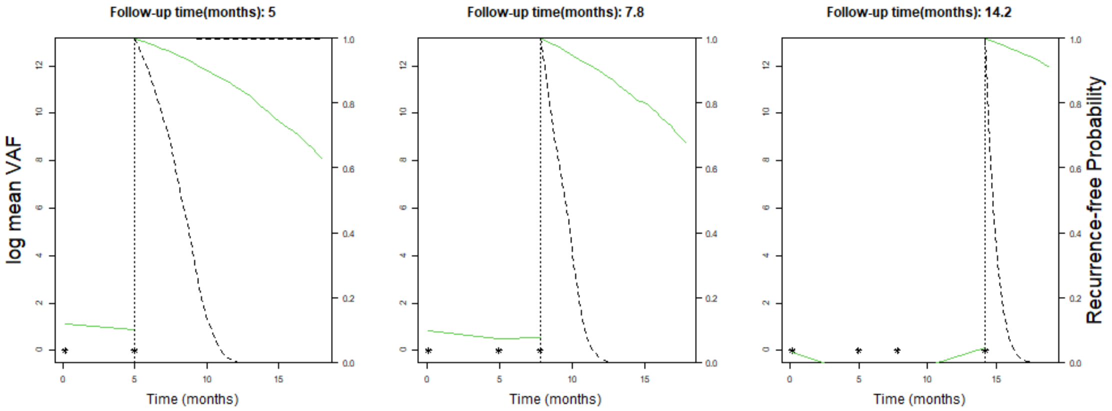

P068, Relaspe-free

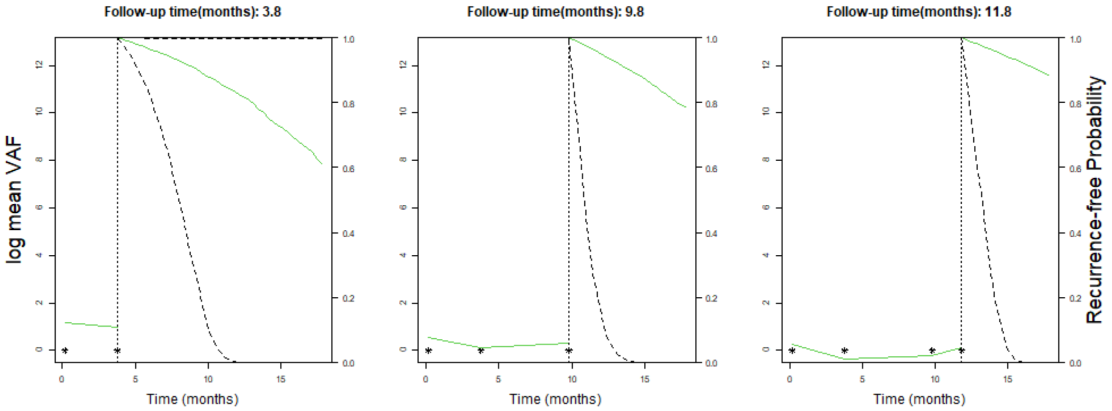

P069, Relapsed

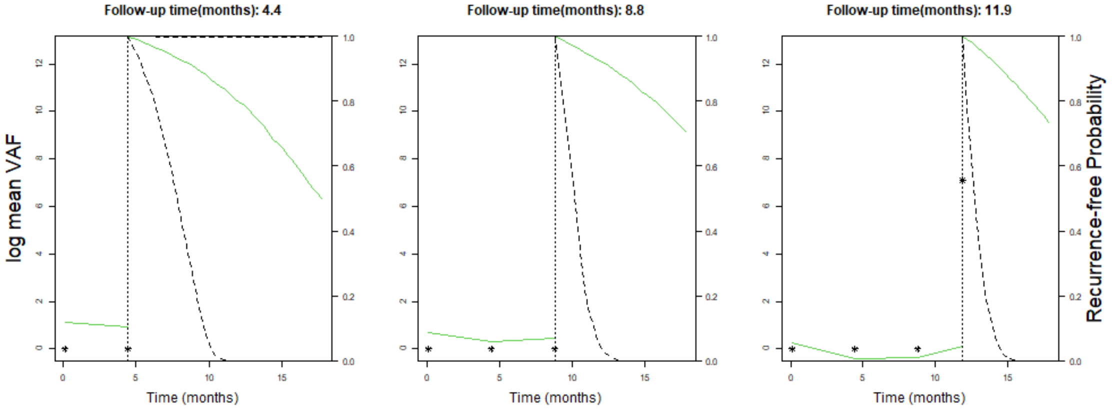

P070, Relaspe-free

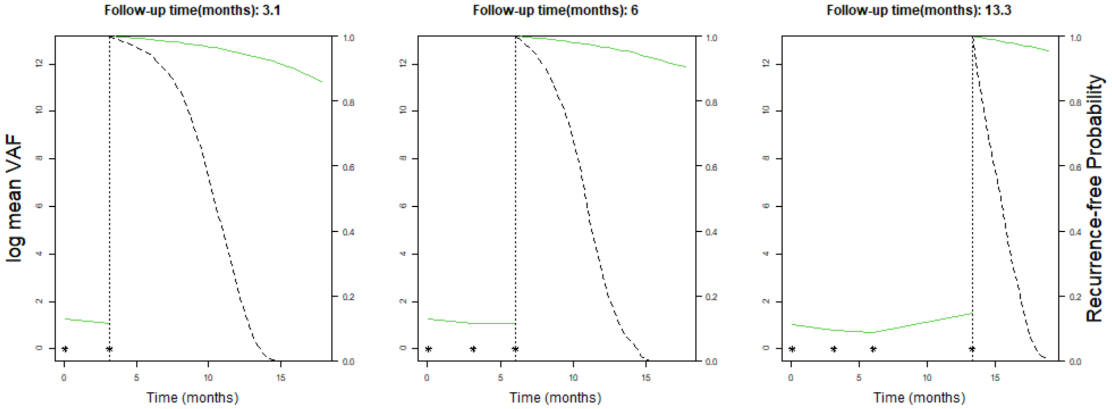

P071, Relapsed

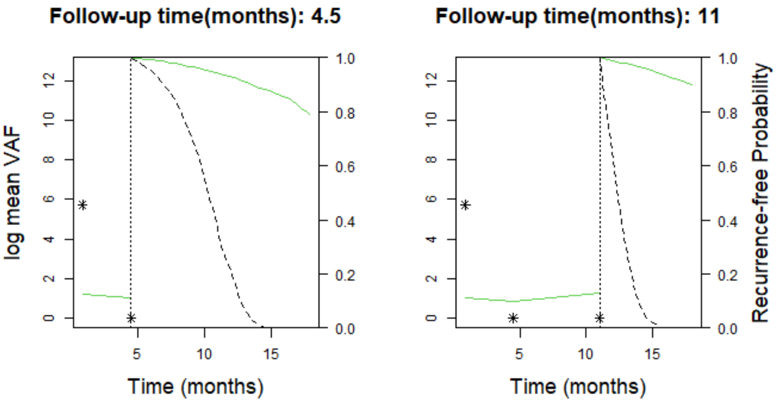

P072, Relapse-free

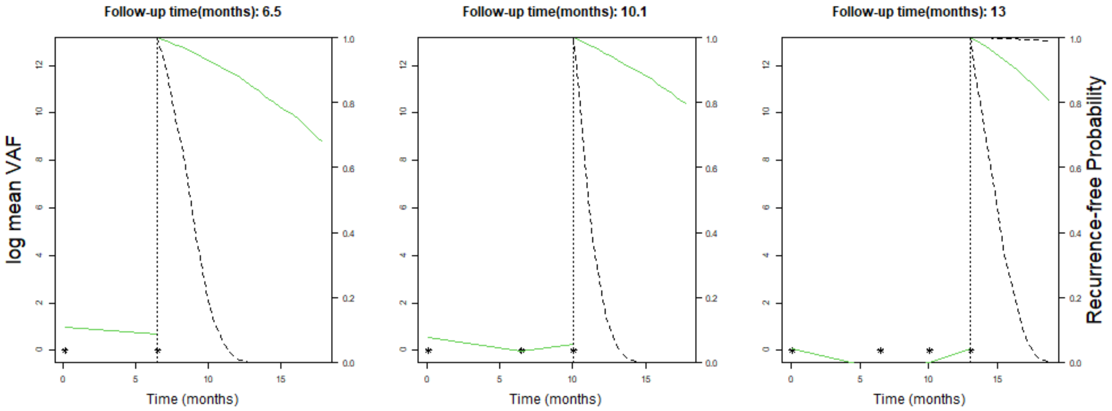

P073, Relaspe-free

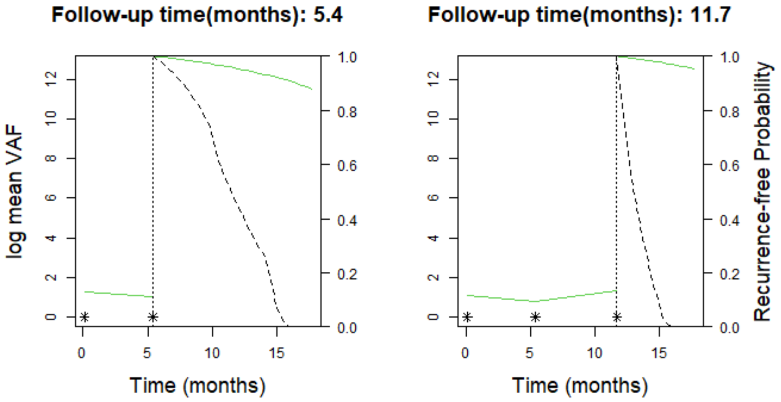

P074, Relapsed

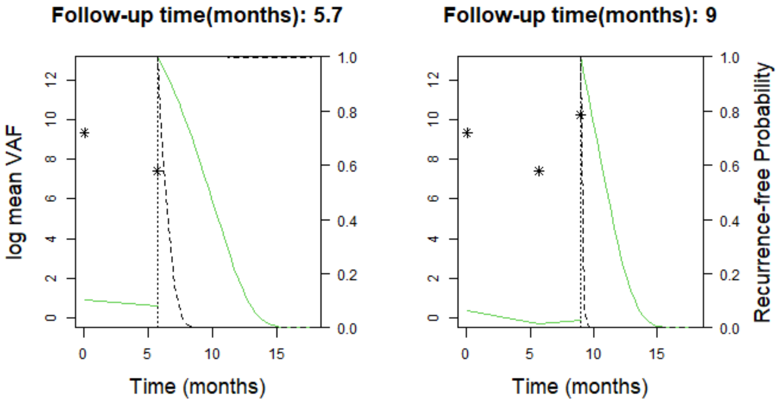

P075, Relaspe-free

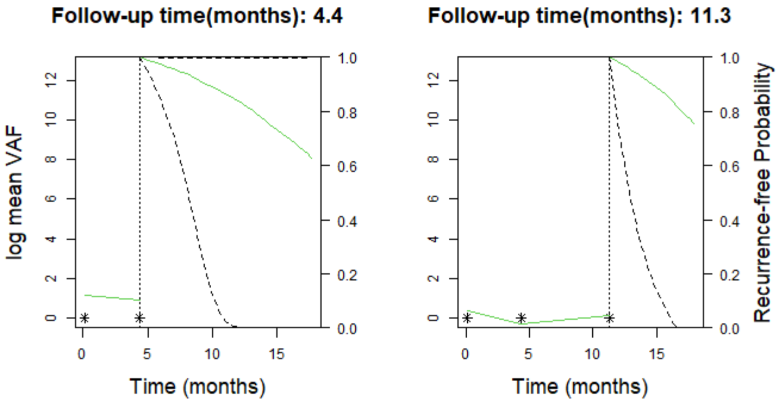

P076, Relaspe-free

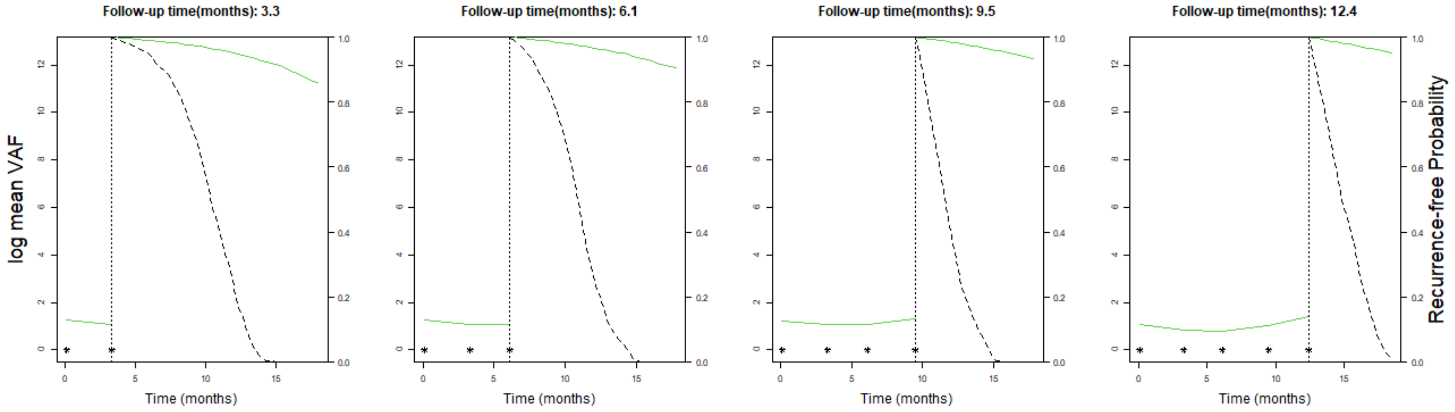

P077, Relapsed

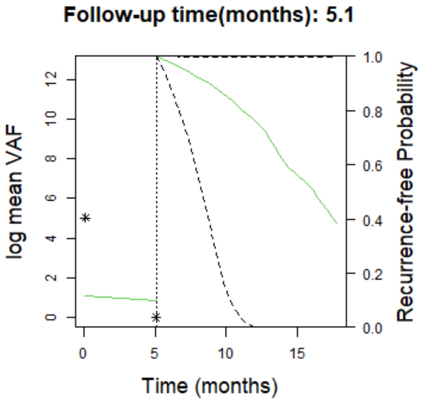

P078, Relaspe-free

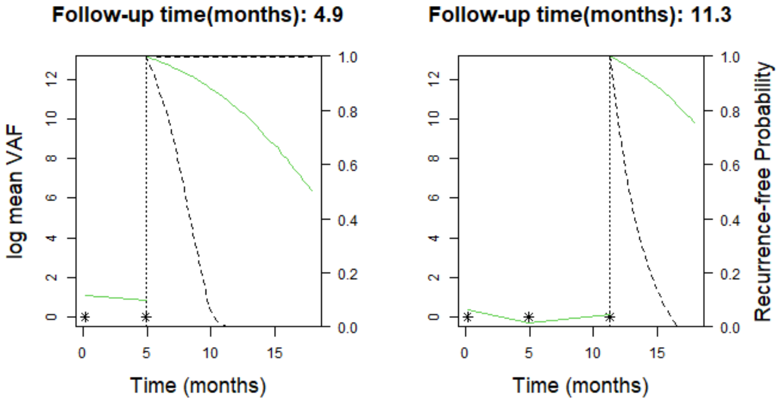

P079, Relapsed

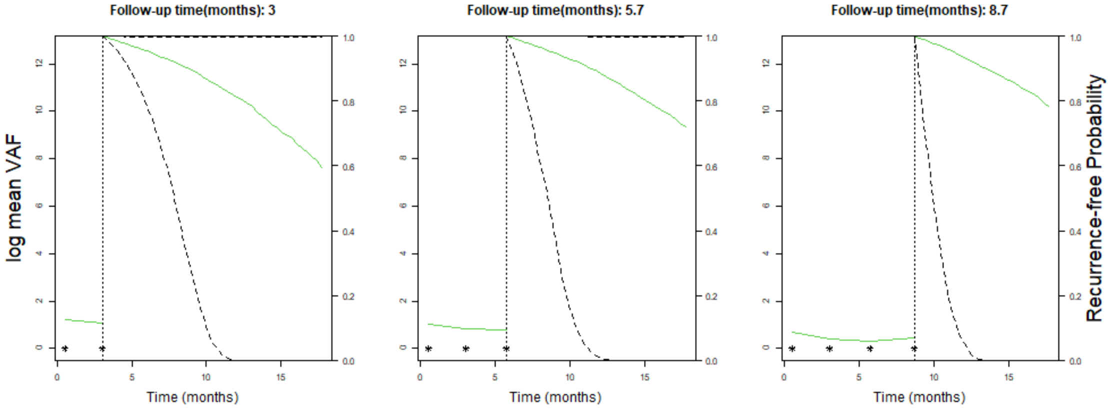

P080, Relaspe-free

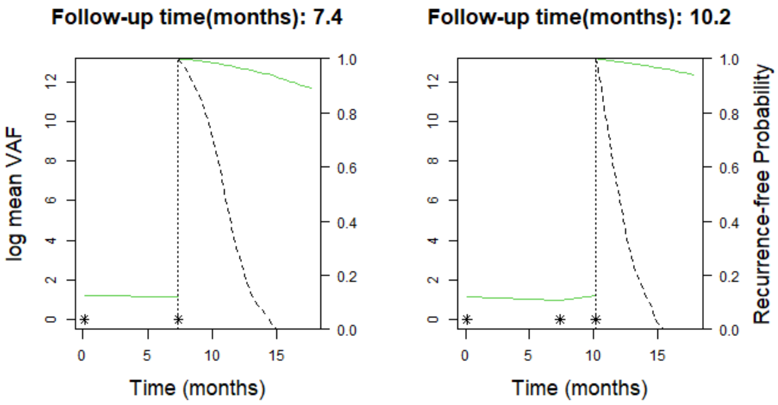

P081, Relapsed

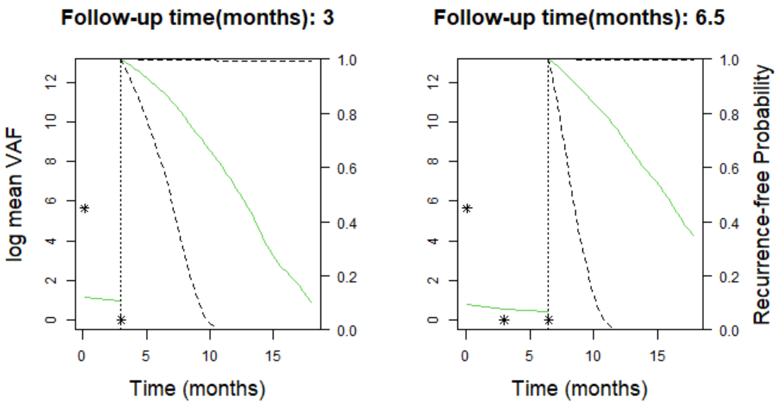

P082, Relapsed

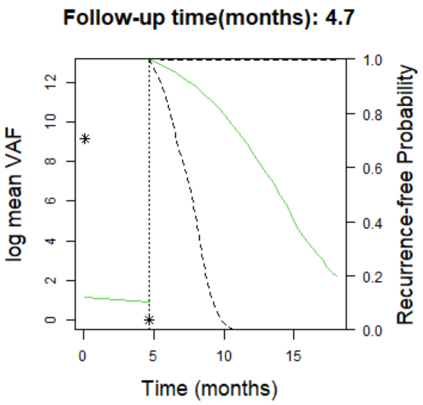

P083, Relapsed

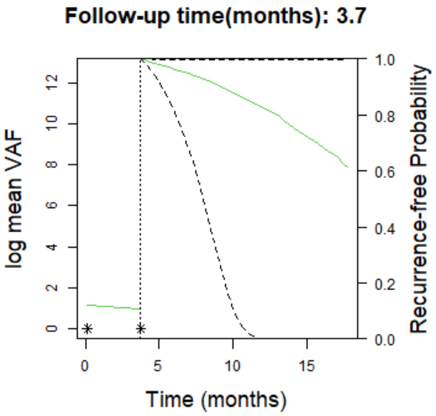

P084, Relaspe-free

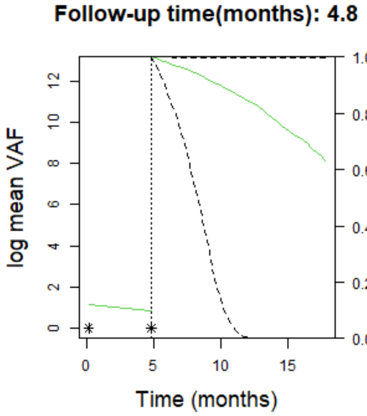

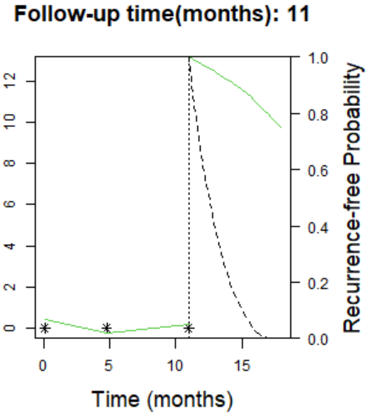

P085, Relaspe-free

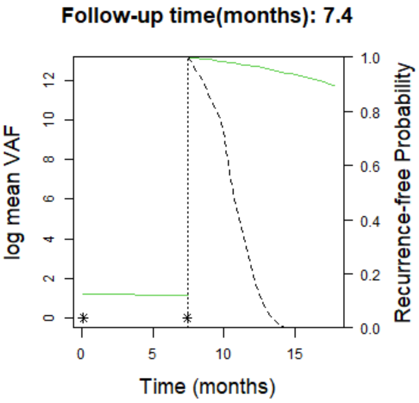

P086, Relaspe-free

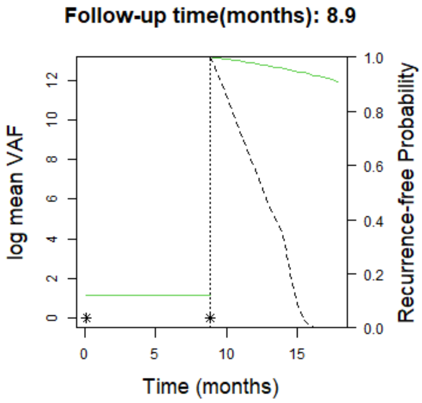

P087, Relapsed

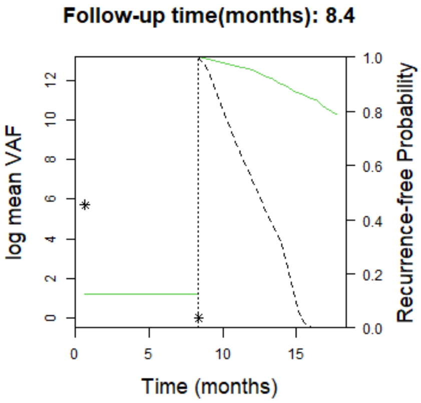

P089, Relaspe-free

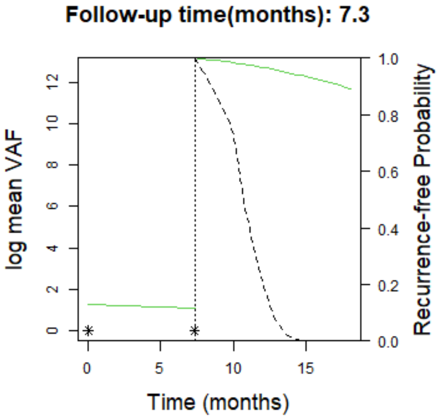

P091, Relaspe-free

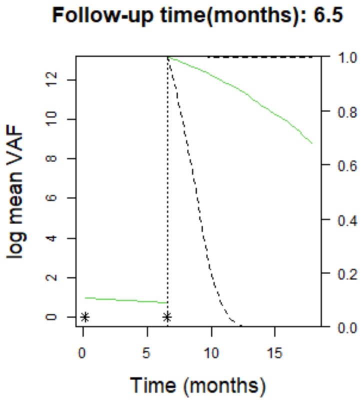

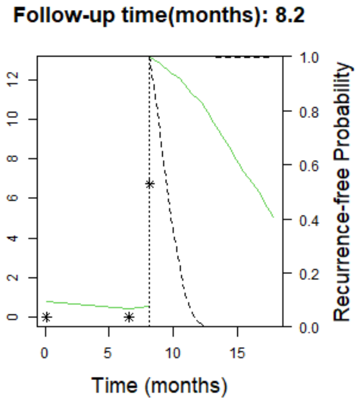

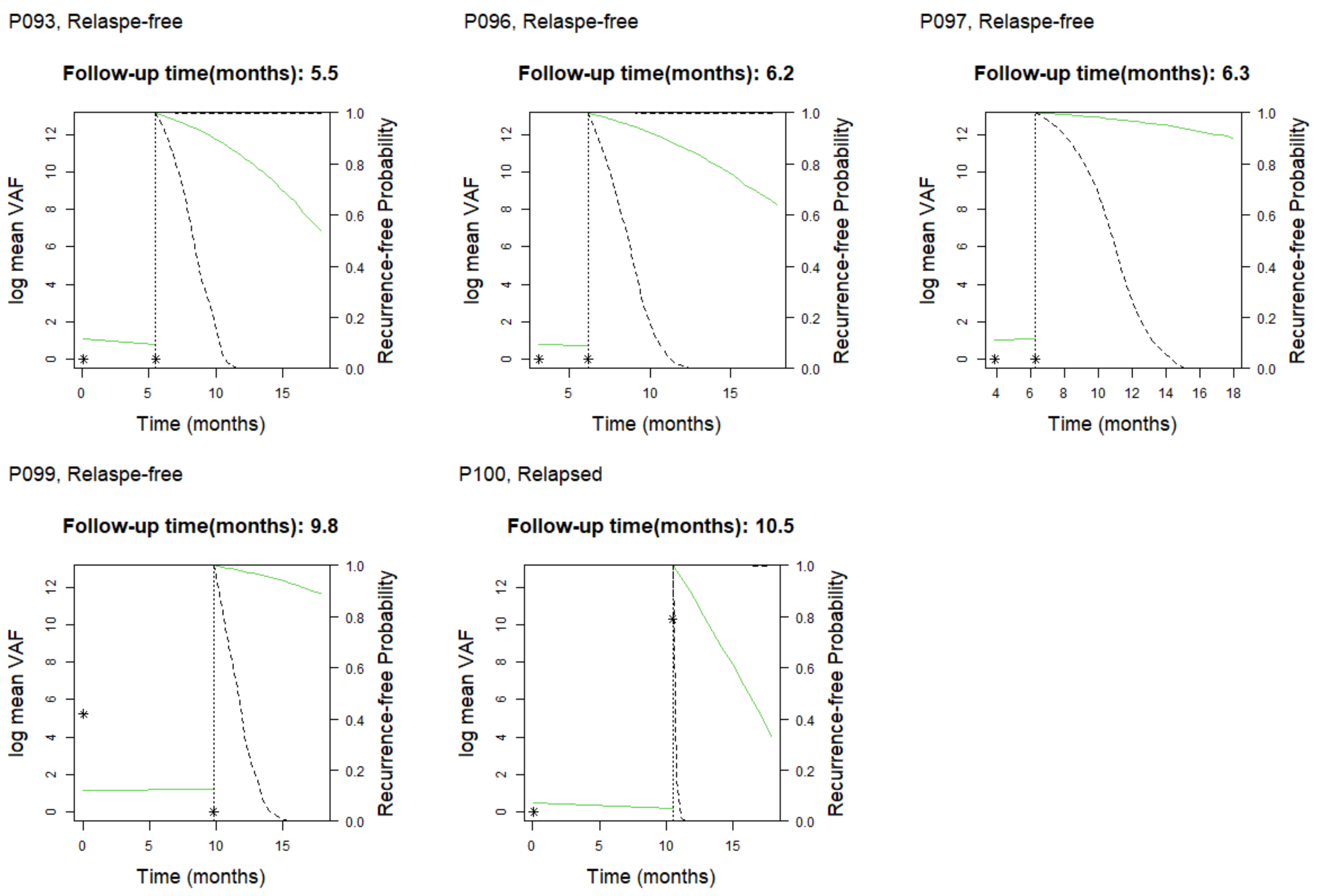

Supplementary Fig. 13 Personalized dynamic risk prediction for patients with two or more blood collection. The recurrence-free probability curve did not show large changes for non-relapsed patients and relapsed patients had considerable decline in the recurrence-free probability. The vertical dotted lines represent the time point of the last ctDNA measurement. To the left of the vertical line is fitted longitudinal trajectory. To the right of the vertical line is the median estimator for event-free probability with 95% pointwise uncertainty band. $Log\ mean\ VAF\ = \ln(mean\ VAF + 10^{-6}) - \ln 10^{-6}$.

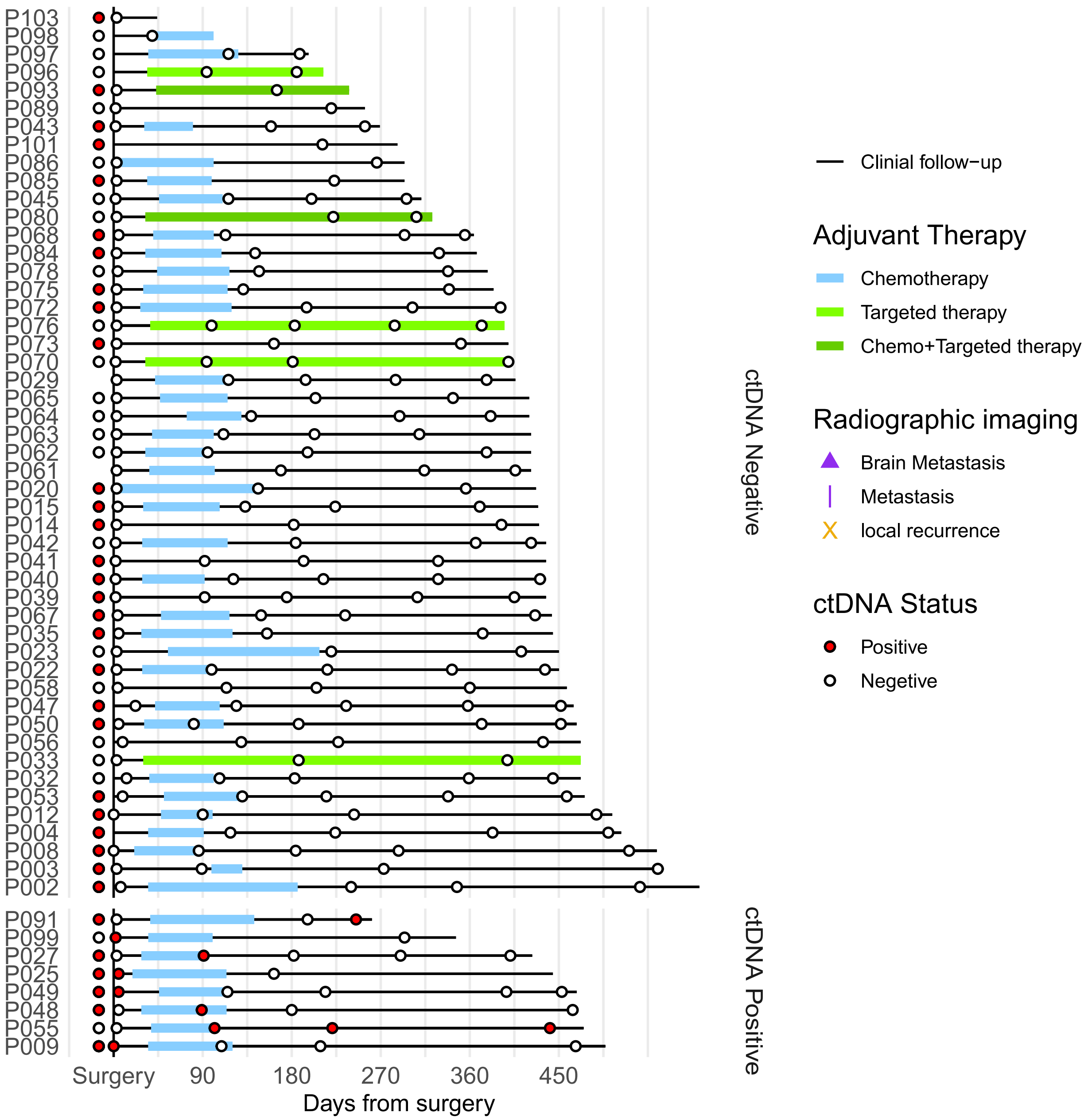

Supplementary Fig. 14 Longitudinal ctDNA results in patients without radiological recurrence. Circles represented ctDNA status. Treatment and imaging information was indicated for each patient. Patients were separated based on their longitudinal ctDNA shedding status.

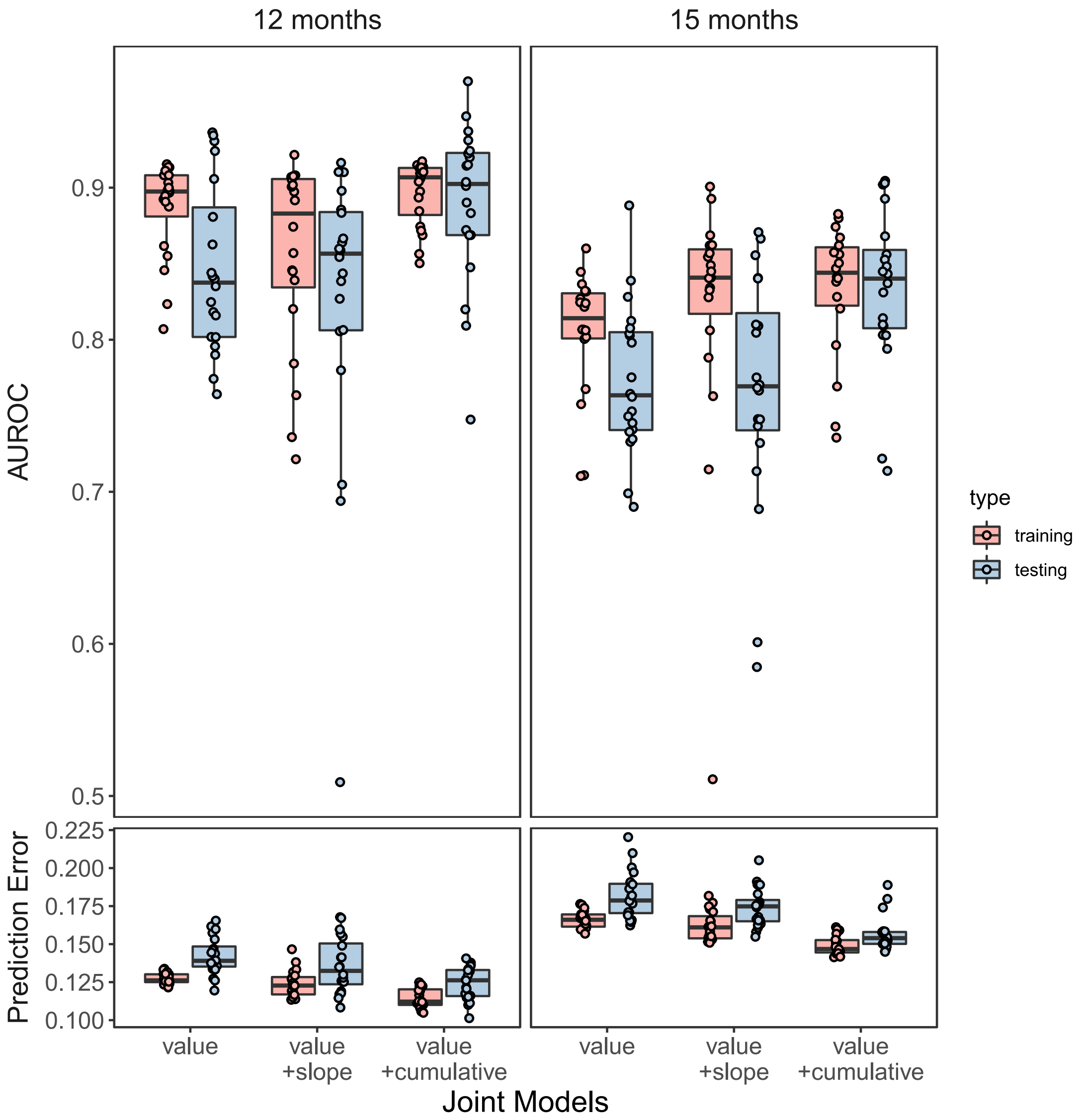

Supplementary Fig. 15 The comparison of model performance between the joint model using different association assumptions. Five-fold cross validation were repeated for 20 times. Time-dependent areas under the receiver-operating characteristics curves (AUROC) and prediction error (PE) represent discrimination power and calibration of the models. Center line, median; box limits, upper and lower quartiles; whiskers, 1.5x interquartile range.
