# Supplementary material for: Dynamic recurrence risk and adjuvant chemotherapy benefit prediction by ctDNA in resected NSCLC
Source: Nat Commun. 2021 Nov 19;12:6770. doi: 10.1038/s41467-021-27022-z (PMC8605017; doi:10.1038/s41467-021-27022-z)
Supplement: Supplementary file 3 — Description of Additional Supplementary Files [file 41467_2021_27022_MOESM3_ESM.docx]

Description of Additional Supplementary Files

Title: Supplementary Data 1

Description: Patient, treatment and tumor baseline characteristics.

Title: Supplementary Data 2

Description: Variant-level data for all tumor reporters at all time points.

Title: Supplementary Data 3

Description: Plasma DNA metrics at each time point for patients.
