# Supplementary material for: Dynamic recurrence risk and adjuvant chemotherapy benefit prediction by ctDNA in resected NSCLC
Source: Nat Commun. 2021 Nov 19;12:6770. doi: 10.1038/s41467-021-27022-z (PMC8605017; doi:10.1038/s41467-021-27022-z)
Supplement: Supplementary file 7 — Reporting Summary [file 41467_2021_27022_MOESM7_ESM.pdf]

nature research

Corresponding author(s): Shugeng Gao

Last updated by author(s): Sep 27, 2021

Reporting Summary

Nature Research wishes to improve the reproducibility of the work that we publish. This form provides structure for consistency and transparency in reporting. For further information on Nature Research policies, see our Editorial Policies and the Editorial Policy Checklist.

Statistics

For all statistical analyses, confirm that the following items are present in the figure legend, table legend, main text, or Methods section.

n/a	Confirmed	
☐	☒	The exact sample size (*n*) for each experimental group/condition, given as a discrete number and unit of measurement
☐	☒	A statement on whether measurements were taken from distinct samples or whether the same sample was measured repeatedly
☐	☒	The statistical test(s) used AND whether they are one- or two-sided<br>*Only common tests should be described solely by name; describe more complex techniques in the Methods section.*
☐	☒	A description of all covariates tested
☒	☐	A description of any assumptions or corrections, such as tests of normality and adjustment for multiple comparisons
☐	☒	A full description of the statistical parameters including central tendency (e.g. means) or other basic estimates (e.g. regression coefficient) AND variation (e.g. standard deviation) or associated estimates of uncertainty (e.g. confidence intervals)
☐	☒	For null hypothesis testing, the test statistic (e.g. *F*, *t*, *r*) with confidence intervals, effect sizes, degrees of freedom and *P* value noted<br>*Give P values as exact values whenever suitable.*
☒	☐	For Bayesian analysis, information on the choice of priors and Markov chain Monte Carlo settings
☒	☐	For hierarchical and complex designs, identification of the appropriate level for tests and full reporting of outcomes
☒	☐	Estimates of effect sizes (e.g. Cohen's *d*, Pearson's *r*), indicating how they were calculated

Our web collection on statistics for biologists contains articles on many of the points above.

Software and code

Policy information about availability of computer code

Data collection	No software are used for data collection.
Data analysis	Trimmomatic was used for FASTQ file quality control. Qualified reads were then mapped to reference human genome (hg19) using Burrows-Wheeler Aligner. PCR duplicates were removed by Picard (Broad Institute, MA, USA) after local realignment around known indels and base quality recalibration using Genome Analysis Toolkit (GATK 3.4.0). Single-nucleotide variations and insertion/deletion were detected using VarScan2. Genomic fusions were identified by FACTERA. All statistical analyses were performed using R version 4.0.2. R package 'survival' (version 3.2-10) was used for survival analysis. R package 'JMbayes' (version 0.8-85) was used for construction and evaluation of joint models and cox models. Reference scripts to reproduce the results of this study is available at https://github.com/cancer-oncogenomics/ctDNA-dynamic-prediction-lung-cancer.

For manuscripts utilizing custom algorithms or software that are central to the research but not yet described in published literature, software must be made available to editors and reviewers. We strongly encourage code deposition in a community repository (e.g. GitHub). See the Nature Research guidelines for submitting code & software for further information.

Data

Policy information about availability of data

All manuscripts must include a data availability statement. This statement should provide the following information, where applicable:

- Accession codes, unique identifiers, or web links for publicly available datasets
- A list of figures that have associated raw data
- A description of any restrictions on data availability

All raw targeted DNA-sequencing data have been deposited in the National Genomics Data Center (NGDC) under the accession code HRA001346 (https://bigd.big.ac.cn/gsa-human/browse/HRA001346). The deposited and publicly available data are compliant with the regulations of the Ministry of Science and

Technology of the People's Republic of China. The raw sequencing data contain information unique to individuals and are available under controlled access. Access to the data can be requested by completing the application form via GSA-Human System and is granted by the corresponding Data Access Committee. Additional guidance can be found at the GSA-Human System website [https://ngdc.cncb.ac.cn/gsa-human/document/GSA-Human_Request_Guide_for_Users_us.pdf]. Data used for survival analysis and joint model construction and evaluation are publicly available at https://github.com/cancer-oncogenomics/ctDNA-dynamic-prediction-lung-cancer. All specific mutation genomic locations and allele frequencies are available in Supplementary Data 2. Source data are provided with this paper.

Field-specific reporting

Please select the one below that is the best fit for your research. If you are not sure, read the appropriate sections before making your selection.

- [x] Life sciences
- [ ] Behavioural & social sciences
- [ ] Ecological, evolutionary & environmental sciences

For a reference copy of the document with all sections, see nature.com/documents/nr-reporting-summary-flat.pdf

Life sciences study design

All studies must disclose on these points even when the disclosure is negative.

Sample size	This study included 397 plasma samples from 103 patients with resectable NSCLC. Sample size was not predetermined based on statistical methods, but was chosen on the basis of prior studies that showed significant effects with similar sample sizes (Abbosh, Christopher, et al. Nature 545.7655 (2017): 446-451; Chaudhuri, Aadel A., et al. Cancer discovery 7.12 (2017): 1394-1403.).
Data exclusions	13 patients who lost to follow up, withdrew consent, and with SCLC or with non-cancer causes of death were excluded.
Replication	This study is a cohort study and therefore is not applicable for experimental replication.
Randomization	There is no randomization as part of this study. Patients were enrolled based on their cancer types and were not allocated into experimental groups.
Blinding	Investigators who collected and processed samples were blinded to survival outcomes while conducting the ctDNA measurements. Blinding was not applicable for analyses involving comparison of patient groups based on their clinical outcomes.

Reporting for specific materials, systems and methods

We require information from authors about some types of materials, experimental systems and methods used in many studies. Here, indicate whether each material, system or method listed is relevant to your study. If you are not sure if a list item applies to your research, read the appropriate section before selecting a response.

Materials & experimental systems

n/a	Involved in the study	
[x]	[ ]	Antibodies
[x]	[ ]	Eukaryotic cell lines
[x]	[ ]	Palaeontology and archaeology
[x]	[ ]	Animals and other organisms
[ ]	[x]	Human research participants
[ ]	[x]	Clinical data
[x]	[ ]	Dual use research of concern

Methods

n/a	Involved in the study	
[x]	[ ]	ChIP-seq
[x]	[ ]	Flow cytometry
[x]	[ ]	MRI-based neuroimaging

Human research participants

Policy information about studies involving human research participants

Population characteristics	Median age: 64 yeas (range from 38 to 82). Sex: 35% females. Smoking status: 59% smoking.
Recruitment	Patients in this study were recruited at the Cancer Hospital of Chinese Academy of Medical Sciences from 2018 to 2020 (ChiCTR1900024656). Patients who were aged at >=18 years old (including both males and females) and with resectable non-small cell lung cancer confirmed by histology and/or cytology were included. Potential self-selection bias or other biases were not identified.
Ethics oversight	The study was approved by the Ethics Committee of Cancer Hospital, Chinese Academy of Medical Sciences and Peking Union Medical College. All patients provided oral and written informed consent.

Note that full information on the approval of the study protocol must also be provided in the manuscript.

Clinical data

Policy information about clinical studies

All manuscripts should comply with the ICMJEguidelines for publication of clinical research and a completedCONSORT checklist must be included with all submissions.

Clinical trial registration	This study was registered at Chinese Clinical Trial Registry (ChiCTR) (ChiCTR1900024656; data of registration 20/07/2019).
Study protocol	Study protocol will be available with publication.
Data collection	Patients were enrolled at Cancer Hospital Chinese Academy of Medical Sciences from 2018 to 2020. Tumor tissues were collected at surgery and pretreatment blood samples were collected before surgery. The first post-surgical blood samples were collected within 30 days after surgery. Patients were then scheduled to be followed every 3 months with computed tomography scan and blood collections until recurrences determined by computed tomography (CT) scan results.
Outcomes	Primary outcome was recurrence measured by the CT imaging diagnostic result. Secondary outcome was ctDNA mutations measured by the NGS analysis.
